# Evidence for Divisome Localization Mechanisms Independent of the Min System and SlmA in *Escherichia coli*

**DOI:** 10.1371/journal.pgen.1004504

**Published:** 2014-08-07

**Authors:** Matthew W. Bailey, Paola Bisicchia, Boyd T. Warren, David J. Sherratt, Jaan Männik

**Affiliations:** 1 Department of Physics and Astronomy, University of Tennessee, Knoxville, Tennessee, United States of America; 2 Department of Biochemistry, University of Oxford, Oxford, United Kingdom; 3 Department of Microbiology, University of Tennessee, Knoxville, Tennessee, United States of America; 4 Department of Biochemistry and Cellular and Molecular Biology, University of Tennessee, Knoxville, Tennessee, United States of America; University of Geneva Medical School, Switzerland

## Abstract

Cell division in *Escherichia coli* starts with assembly of FtsZ protofilaments into a ring-like structure, the Z-ring. Positioning of the Z-ring at midcell is thought to be coordinated by two regulatory systems, nucleoid occlusion and the Min system. In *E. coli*, nucleoid occlusion is mediated by the SlmA proteins. Here, we address the question of whether there are additional positioning systems that are capable of localizing the *E. coli* divisome with respect to the cell center. Using quantitative fluorescence imaging we show that slow growing cells lacking functional Min and SlmA nucleoid occlusion systems continue to divide preferentially at midcell. We find that the initial Z-ring assembly occurs over the center of the nucleoid instead of nucleoid-free regions under these conditions. We determine that Z-ring formation begins shortly after the arrival of the Ter macrodomain at the nucleoid center. Removal of either the MatP, ZapB, or ZapA proteins significantly affects the accuracy and precision of Z-ring positioning relative to the nucleoid center in these cells in accordance with the idea that these proteins link the Ter macrodomain and the Z-ring. Interestingly, even in the absence of Min, SlmA, and the putative Ter macrodomain – Z-ring link, there remains a weak midcell positioning bias for the Z-ring. Our work demonstrates that additional Z-ring localization systems are present in *E. coli* than are known currently. In particular, we identify that the Ter macrodomain acts as a landmark for the Z-ring in the presence of MatP, ZapB and ZapA proteins.

## Author Summary

Cell division in *Escherichia coli* begins with the assembly of FtsZ proteins into a ring-like structure, the Z-ring. Remarkably, the Z-ring localizes with very high precision at midcell. Currently, two molecular systems, nucleoid occlusion and the Min system, are known to localize the Z-ring. Here, we explore whether there are additional divisome localization systems in *E. coli*. Using quantitative fluorescence imaging, we show that slow growing cells lacking both known positioning systems continue to divide accurately at midcell. We find that the terminus region of the chromosome moves first to mid-cell where it functions as a positional landmark for the subsequent localization of the Z-ring. Furthermore, we provide evidence that this divisome positioning system involves MatP, ZapB, and ZapA proteins. Our work shows that *E. coli* can divide without the canonical mechanisms for localizing its cytokinetic ring. In particular, we identify that the Ter macrodomain acts as a landmark for the Z-ring in the presence of MatP, ZapB and ZapA proteins.

## Introduction

Cell division is an essential cellular process that requires accurate spatial and temporal positioning of cytokinetic proteins. Assembly of the cell division apparatus, the divisome, must be coordinated closely with replication and segregation of chromosomes to ensure that each daughter cell receives an integral genome from the mother. The assembly of the divisome in *Escherichia coli* starts with the formation of a macromolecular structure, called the Z-ring, which encircles the rod-shaped cell in its geometric middle [1]–[4]. The Z-ring consists of filaments of FtsZ proteins, which are anchored to the cell membrane through the FtsA and ZipA linker proteins. The Z-ring serves as a scaffold for more than a dozen other divisome proteins, which build the cell envelope between the two daughters and mediate partitioning of the chromosomes into newly forming compartments [5].

In *E. coli*, the divisome is positioned at the cell center with remarkable accuracy [6]–[9]. How do nanometer-scale FtsZ proteins recognize the center of the cell with such high accuracy and at the same time provide faithful coordination between the divisome and the chromosome? The current view holds that Z-ring localization is governed by two independent mechanisms in *E. coli*, the Min system and nucleoid occlusion [2]–[4], [10] that both negatively regulate Z-ring polymerization. The Min system is composed of the MinC, MinD, and MinE proteins that together exhibit dynamic pole-to-pole oscillation [11]. While the MinD and MinE proteins are essential for such oscillation, MinC acts as the sole inhibitor of Z-ring formation by binding to FtsZ [12]. Considering that the minimum of the time-averaged concentration of MinC occurs at midcell, the Min system protects cell poles from developing septa and guides localization of the Z-ring to the center of the cell [13].

The nucleoid occlusion mechanism was first proposed on a phenomenological level to account for a lack of division septa from forming over the nucleoid [14], [15]. It has been established that the SlmA protein mediates nucleoid occlusion in *E. coli* [16], while a similar factor, the Noc protein, was found in *Bacillus subtilis* [17]. The two proteins do not share sequence similarity but they apparently function in a similar manner. Both SlmA [18]–[20] and Noc [21] are DNA-binding proteins that are capable of inhibiting Z-ring formation in their DNA-bound form. SlmA and Noc lack binding sites in the vicinity of the replication terminus (Ter). Such positioning assures that their Z-ring inhibiting activity is relieved at midcell when two daughter chromosomes segregate.

*E. coli* cells that lack both the Min system and SlmA are not capable of dividing in rich LB medium, instead forming long filamentous cells [16]. Although this finding could imply that the Min system and SlmA are the only localization systems for the divisome in *E. coli*, it was found that the same cells can grow and divide in nutrient poor M9 medium, and even in LB when FtsZ levels were artificially upregulated [16]. It was also found that deletion of SlmA alone did not cause any loss in cell division accuracy, and the correlations between the divisome and chromosome localizations remained the same in these cells compared to wild type [6]. These findings imply that there exists a SlmA-independent mechanism that localizes cell division proteins relative to chromosomes in *E. coli*. Similar to *E. coli*, evidence of Noc-independent nucleoid occlusion exists in *B. subtilis* [22], [23]. Moreover, it was found that *B. subtilis* cells were capable of positioning the Z-rings precisely at midcell in the complete absence of any nucleoid occlusion and the Min system [24]. These findings warrant revisiting the canonical model that the Min system and SlmA/Noc mediated nucleoid occlusion together are the sole factors coordinating the localization of cell division proteins in bacteria, and raise the question of what additional mechanisms bacterial cells use to position their divisome.

Here, we study cell division in *E. coli* strains lacking both the Min system and nucleoid occlusion factor SlmA to identify new mechanisms involved in Z-ring localization. We use high-resolution quantitative fluorescence imaging to resolve nanometer-scale changes in positions of the Z-rings and cell division planes. We show that Min and SlmA double deletion cells are capable of accurately localizing their division planes in slow growth conditions. In this process, *E. coli* frequently positions its Z-ring initially over the nucleoid center instead of at nucleoid-free regions. We determine that during the formation of the Z-ring, the nucleoid center is occupied by the Ter macrodomain region of the chromosome. MatP, ZapB, and ZapA proteins, which have been implicated in linking the Ter macrodomain and the Z-ring [25], affect the accuracy and the precision of the Z-ring positioning relative to the nucleoid center. However, *E. coli* Δ*slmA* Δ*min* cells without MatP, ZapB, and ZapA are still capable of positioning their Z-rings close to the cell centers, albeit with lower precision.

## Results

### *ΔslmA Δmin* cells divide at well-defined locations relative to cell poles

**Figure 1:**
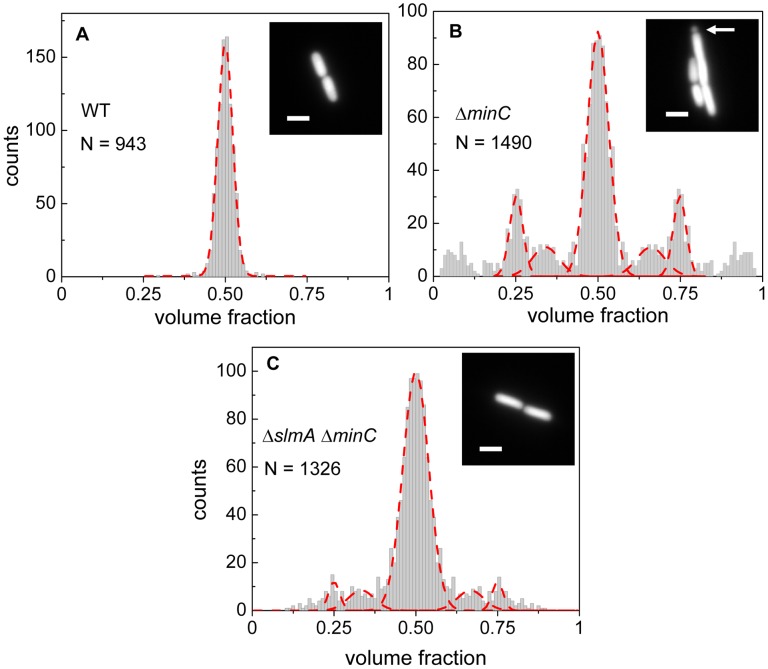
Relative volume fractions of daughter cells after division. (A) Wild type (BW25113), (B) Δ*minC* (JW1165), (C) Δ*slmA* Δ*minC* (PB194) strains. Volume fractions are calculated as the ratio of one daughter cell's volume to the sum of both daughters' volumes. Red dashed lines in the histogram show fittings of different peaks with a Gaussian function. The centers of fitting lines are fixed to 1/4, 1/3, 1/2, 2/3 and 3/4 values. The insets in the histograms show fluorescent images of cells from the respective strains. The arrow in the inset of panel (B) points to a minicelling division. All scale bars correspond to 2 µm.

Details about how cell division occurs in *E. coli* Δ*slmA* Δ*min* strain has not yet been described in slow growth conditions where cells are capable of dividing and propagating. One would expect that if SlmA-mediated nucleoid occlusion and the Min system are the only two positioning systems in *E. coli*, then the division planes in these double mutant cells should be localized completely randomly. Surprisingly, we found this not to be the case. The majority of Δ*slmA* Δ*minC* cells appeared to divide about the cell center, and all the cells retained normal morphology in minimal M9 medium. To quantify the accuracy of division plane placement in these cells, we determined the relative volume fractions of two daughter cells that still adhere together by their poles after the division and compiled these ratios into a histogram (Figure 1). To calculate the volume fractions, we used fluorescent images of cells, which carried a cytosolic GFP label, and applied a quantitative image analysis procedure as described earlier [6]. As a reference, we determined the volume fraction distributions for the parental strain/wild type BW25113 (Figure 1A) and strain JW1165 having only a *minC* deletion (Figure 1B). For all the strains used in this work, see Table S1. As expected, the distribution of volume fractions for the parental strain consisted of a pronounced single peak at a value of 1/2, showing that upon division, the volume of each daughter cell is approximately equal. Note that all histograms are symmetric relative to 1/2 because both daughter cells are counted in these histograms. Also as expected, the distribution of volume fractions for the Δ*minC* strain showed distinct peaks at 1/4, 1/3, 2/3 and 3/4 values, in addition to the main peak at 1/2. All these peaks arise because of underlying nucleoid structure. Peaks at 1/4 and 3/4 values correspond to divisions where a mother cell distributes one of its nucleoids to one daughter cell and three to the other. Smaller peaks at 1/3 and 2/3 values correspond to division of cells with three nucleoids. The Δ*minC* strain also showed minicelling divisions which appeared as broad peaks on the tails of the volume fraction histogram.

The volume fraction distribution for the Δ*slmA* Δ*minC* strain (PB194) showed, qualitatively similar to the Δ*minC* strain, distinct peaks at 1/2, 1/4 and 3/4 positions with discernible peaks also at 1/3 and 2/3 values (Figure 1C). Gaussian fits to the peaks in the histogram showed that the majority of Δ*slmA* Δ*minC* cells divide at about midcell (75%) while 6.5% divided approximately at the quarter position, and 14% between the quarter and half-cell length from one of the poles. Interestingly, the frequency of central divisions for the Δ*slmA* Δ*minC* strain was higher than for the strain having only a *minC* deletion (50%) while the frequency of *ΔminC* cells dividing at a quarter (20%) and a third of the cell length from the poles (16%) was higher compared to the Δ*slmA* Δ*minC* strain. Additionally, the double mutant strain produced essentially no minicells (0.2% of total divisions), although they were noticeably present in the *minC* deletion strain (7% of total divisions). The presence of peaks at the 1/2, 1/4, and 3/4 positions indicates that, despite a lack of nucleoid occlusion factor SlmA in the double mutant strain, there remains a high level of coordination between nucleoids and the Z-rings in *E. coli* cells. Comparison between Δ*minC* Δ*slmA* double mutants and Δ*minC* single mutant strains further shows that removal of SlmA suppresses minicell production and biases cell division towards the cell center.

### The cell division plane shifts from midcell to quarter-cell with increasing cell length

**Figure 2:**
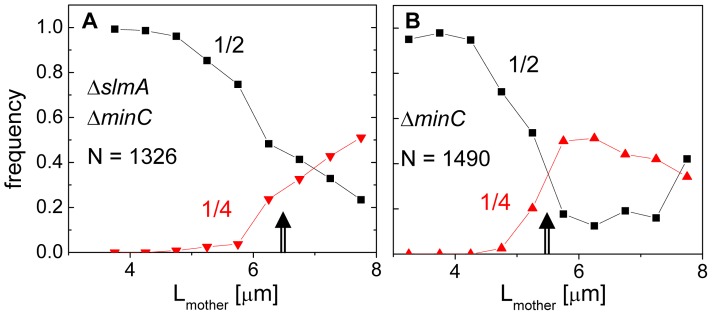
Division frequency at the 1/4 and 1/2 cell positions with respect to mother cell length. (A) Data for the Δ*slmA* Δ*minC* double mutant strain (PB194); (B) Δ*minC* strain (JW1165). Cell lengths are binned at 0.25 µm intervals. Arrows point to transition regions from centrally occurring divisions to divisions at cell quarters. The lengths of the mother cells are measured just before cell division. Note that only a few cells from both strains are longer than 8 µm, limiting analysis for longer cells.

The length of the double mutant cells immediately following division, 2.83±1.54 µm, was 18% longer than that of the BW25113 parental strain, 2.41±0.36 µm (Figure S1). The main factor contributing to the length difference was long cells making up about 10% of the Δ*slmA* Δ*minC* population, whose lengths were about twice that of the majority of the population. It was noticeable that these longer double mutant cells divided with higher prevalence at 1/4 and 3/4 positions compared to shorter cells. To quantify this tendency, we plotted the frequency of central divisions and the frequency of divisions at the quarter cell length from the poles as a function of mother cell length (Figure 2). In this analysis, the central divisions were considered to be all divisions in which volume fractions were within 0.50±0.10. The divisions at quarter cell positions were considered when the corresponding volume fraction ratios were within 0.25±0.05. For the Δ*slmA* Δ*minC* cells, the frequency of cell divisions at the quarter cell length from the poles increased considerably as the cells reached a length of about 6 µm (Figure 2A). About 50% of cells longer than 6 µm preferentially divided at the quarter-cell length from the poles, while a smaller fraction, about 25% of cells, divided at the cell center. The data for the Δ*minC* cells showed a very similar sharp transition of the cell division plane from the center to the quarter locations as the cell length reached about 5.2 µm (Figure 2B). A marked increase in the frequency of 1/4 divisions indicates that some positional signal guides the cell division plane from midcell to its quarter positions as the cells reach a relatively well-defined length.

### Z-rings localize to the centers of nucleoids

**Figure 3:**
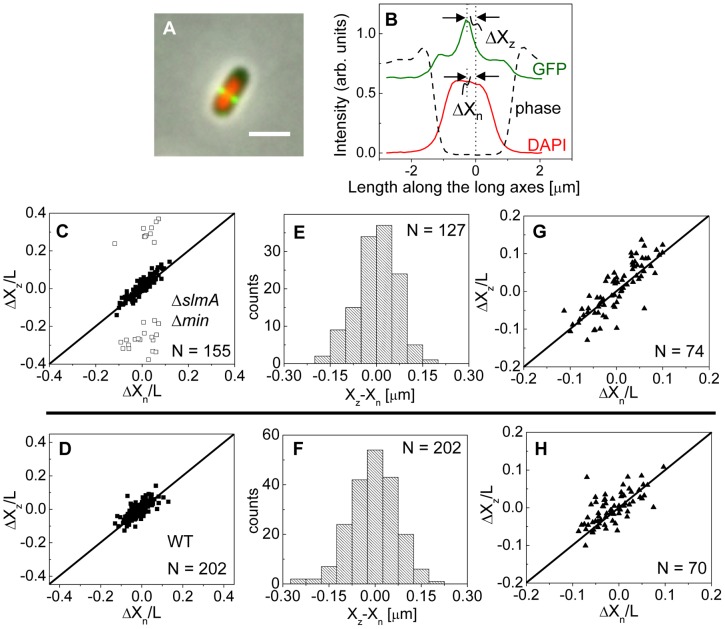
Localization of ZipA-GFP labeled Z-rings relative to cell center and the center of nucleoids. (A) A composite of ZipA-GFP (green), DAPI stained nucleoid (red) and phase contrast images (grey) of a Δ*slmA* Δ*min* cell with a distinctly off-center placed nucleoid. The scale bar is 2 µm. (B) The intensity line profiles of each image plane along the long axis of the cell for the cell shown in panel A. The displacement of the nucleoid relative to the cell center is ΔX_n_, and the displacement of the ZipA-GFP labeled Z-ring is ΔX_z_. (C) ΔX_z_ vs. ΔX_n_ for Δ*slmA* Δ*min* cells (strain TB86) scaled by cell length L. Solid rectangles mark central and open rectangles mark polar Z-rings. The solid line corresponds to 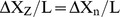. Data are shown only for cells with a single nucleoid. (D) ΔX_z_ vs. ΔX_n_ for the parental strain (strain JMBW5). (E), (F) Distribution of distances between the Z-ring center and nucleoid center for Δ*slmA* Δ*min* strain and parental strain, respectively. Data for central Z-rings are shown. (G), (H) ΔX_z_ vs. ΔX_n_ for cells that show a Z-ring over a compact nucleoid in Δ*slmA* Δ*min* and in parental strain, respectively.

To investigate this positional signal, we determined the placement of the Z-ring relative to the nucleoid and cell centers using the previously described *E. coli* Δ*slmA* Δ*min* double mutant strains with ZipA-GFP (TB86 λCH151) and FtsZ-GFP (TB86 λDR120) labels [16]. In these measurements, the nucleoid was stained with DAPI. As a reference, the parental strain with the same labeling was also imaged. Representative cell images are shown in Figure S2. We noticed that in a few Δ*slmA* Δ*min* cells, the nucleoids were displaced noticeably from the cell center. In these cases, the positions of the Z-rings followed the centers of the nucleoids rather than the centers of the cytosolic volumes (Figure 3A). To quantify the tendency of the Z-ring to localize over the nucleoid center, we measured the distance between the Z-ring and cell center, ΔX_z_, as a function of the distance between the nucleoid center and the cell center, ΔX_n_, for all cells in a population having a single nucleoid (Figure 3B, C). The numerical procedure to determine the centers of the cell, nucleoid, and Z-ring is described in the Text S1. As can be seen from Figure 3B, displacements of nucleoids away from cell centers were associated with correlated displacements of Z-rings. To further analyze the extent of co-localization between the nucleoid center and the Z-ring, we determined the standard deviations of distances between the Z-rings and nucleoid centers, 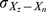, and between the Z-rings and cell centers 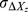. We separated the data into two distinct groups – polar Z-rings and centrally located ones. The precision of central Z-ring placement relative to nucleoid centers, 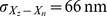 (Figure 3E), was more than two times higher than the positioning of Z-rings relative to cell centers, 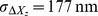, in Δ*slmA* Δ*min* cells with ZipA-GFP label (Figure S3A). We found very similar co-localization characteristics for central Z-rings (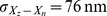, 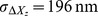) in FtsZ-GFP labeled Δ*slmA* Δ*min* cells (Figure S3B, S4), confirming that the co-localization effect is not related to a specific Z-ring label. The collection of distribution statistics for all measured strains can be found in Table S2. Interestingly, for wild type cells (Figure 3D, F) co-localization between Z-rings and nucleoid centers (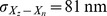) was somewhat lower than in Δ*slmA* Δ*min* cells (Ansari-Bradley test 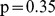; F-test 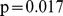), while the precision of Z-ring placement in the vicinity of the cell centers (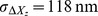) was significantly higher when compared to Δ*slmA* Δ*min* cells (Ansari-Bradley test 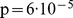; F-test 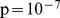). Similar values of 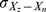 and 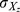 as in wild type cells were found also for Δ*min* and Δ*slmA* single deletion strains (Figure S5; Table S2).

Co-localization of the Z-ring to nucleoid centers was present already in the early stages of chromosomal replication before a distinct bi-lobed morphology appeared in nucleoid images (Figure 3G, H). To distinguish bi-lobed nucleoids from compact nucleoids, we inspected intensity line profiles taken over DAPI stained nucleoids. We considered a nucleoid to be compact if its DAPI intensity line profile near the nucleoid center lacked any discernable dips (e.g. DAPI profile in Figure 3B). In Δ*slmA* Δ*min* cells with a compact nucleoid, the level of co-localization between the nucleoid and the Z-ring, 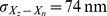, was comparable to the value characterizing the whole cell population, 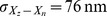 (F-test, 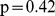; Ansari Bradley test 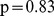). A Similar conclusion can be drawn also for the wild type cells where 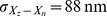 (F-test 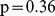; Ansari Bradley test 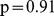) and for Δ*min* and Δ*slmA* single deletion strains (Figure S5; Table S2). These comparisons indicate that nucleoid centers and Z-rings can co-localize in early stages of replication when the nucleoid morphology is compact both in wild type cells and in cells where one or both of the known Z-ring positioning systems have been removed.

**Figure 4:**
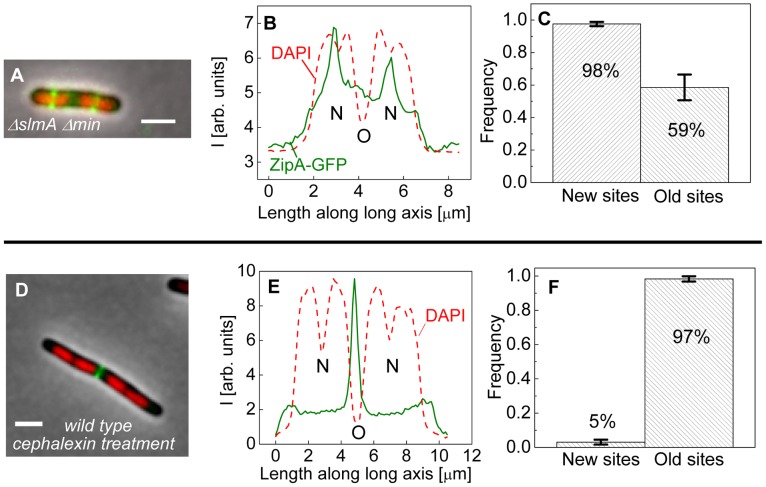
Positioning of Z-rings relative to nucleoids in multi-nucleoid cells. (A) A composite image of longer Δ*slmA* Δ*min* cell. ZipA-GFP (green), DAPI stained nucleoid (red), and phase contrast images (grey) have been overlaid. Scale bar is 2 µm. (B) Nucleoid and ZipA-GFP density distributions along the long axis of the cell for the cell shown in panel (A). The positions marked by “N” correspond to the new division sites at the centers of the nucleoids and the position marked by “O” to old division site between fully segregated nucleoids. (C) Frequency of Z-rings in the double mutant cells at the new and old replication sites. Only cells that have two or more distinct nucleoids have been analyzed. Error bars represent standard deviations over three independent measurements each involving about 50 cells. (D)–(F) the same for wild type cells that have been treated for 2 hours with 20 µg/ml cephalexin.

The bias in localization of the Z-rings to the centers of nucleoids was even more visually striking in longer Δ*slmA* Δ*min* cells that had two or more well-separated nucleoids (Figure 4A). We found a strong preference for the Z-ring to position over the centers of nucleoids as compared to regions between fully segregated nucleoids (Figure 4B). We refer to the former as the new division sites (N) and the latter as the old division sites (O). The probability of finding a Z-ring over the center of nucleoids (N sites) was 98±1%, while the probability of finding a Z-ring in the inter-nucleoid space between fully segregated nucleoids (O sites) decreased to 59±8% (Figure 4C). Note that Z-rings can be present in both division sites at the same time. The tendency of the Z-rings to preferentially localize at ¼ positions from the cell pole in longer cells, i.e. in new sites, is consistent with our earlier observation that in longer Δ*slmA* Δ*min* cells, divisions occur preferentially at ¼ positions from the cell pole (Figure 2). Taken together, the analysis of the placement of the Z-rings and nucleoid centers in multi-nucleoid Δ*slmA* Δ*min* cells further supports the hypothesis that a positional signal guides the Z-rings to the nucleoid centers.

To determine if wild type cells would display the same behavior as multi-nucleoid Δ*slmA* Δ*min* cells we induced an elongated, multi-nucleoid cell morphology by treating cells with cephalexin. Cephalexin does not inhibit Z-ring assembly but prevents Z-ring constriction by inhibiting the downstream protein FtsI (PBP3). Interestingly, in elongated wild type cells the Z-rings appeared essentially only at midcell even when new sites were present (Figure 4 D–F). Z-rings in cephalexin treated Δ*slmA* Δ*minC* cells still showed a preference to the new division sites as did their untreated counterparts (Figure S6 A–C). We also analyzed *ΔslmA* and Δ*minC* single deletion cells after cephalexin treatment. Δ*slmA* cells behaved as wild type cells (Figure S6 D–F) while Z-rings in the Δ*minC* cells showed a preference to the new division sites as in Δ*slmA* Δ*min* cells (Figure S6 G–I). These comparisons show that the putative positioning signal only manifests itself when it is not conflicting the regulation due to the Min system. It is important to note that such conflict does not occur in wild type cells in normal growth conditions because in this case the nucleoid center and the concentration minimum for MinC coincide. As Figures 3 D–H show, the localization signal emanating from the nucleoid center is important in Z-ring localization in wild type cells under normal growth conditions.

### Co-localization between the Ter macrodomain and the Z-ring

**Figure 5:**
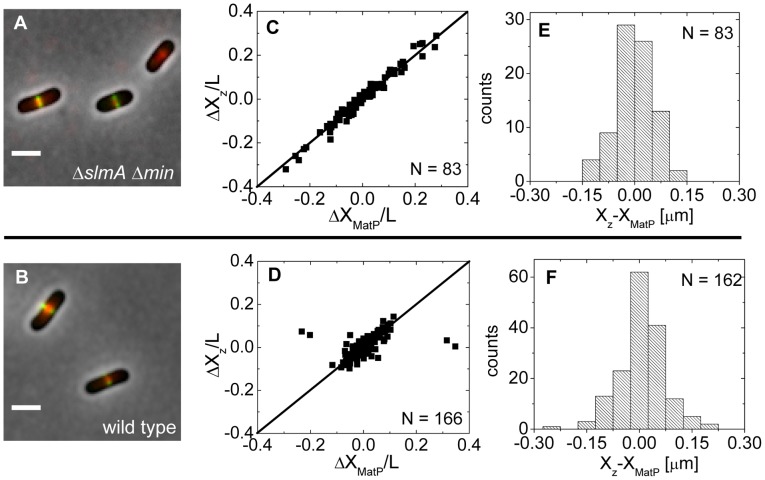
Positioning of the Z-ring relative to the MatP-labeled Ter macrodomain. (A) A composite of ZipA-GFP (green), MatP-mCherry (red), and phase contrast image (grey) of Δ*slmA* Δ*min* cells (strain WD1). Scale bar is 2 µm. (B) The same for the wild type strain (strain WD2). (C) Location of ZipA-GFP labeled Z-ring (ΔX_z_) vs location of MatP-mCherry focus (ΔX_MatP_) in Δ*slmA* Δ*min* cells scaled by the cell length L. Both locations are referenced relative to the cell center. Solid symbols correspond to locations near the center of the nucleoid and open squares to locations near the poles. The straight line corresponds to 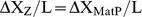. Only cells with a single MatP focus are analyzed. (D) ΔX_z_ vs ΔX_MatP_ for wild type cells. (E), (F) Distribution of distances between the Z-ring and the MatP focus along the long axes of the cell for Δ*slmA* Δ*min* and wild type cells, respectively.

Approximately at the time of Z-ring formation, the center of the nucleoid is known to be occupied by the Ter region of the chromosome [26], [27], which forms a well-defined unit – the Ter macrodomain [28], [29]. In *E. coli*, MatP is a dispensable protein that defines the Ter macrodomain by connecting 23 specific sites in a chromosomal region that spans about 800 kb [28]. Based on previous works [26], [27], it appeared plausible that the Z-rings might position over the Ter macrodomain. To investigate if this hypothesis is correct, we labeled the Ter region of the chromosome with a MatP-mCherry construct that was expressed from its endogenous *matP* locus and we labeled the Z-ring with ZipA-GFP (Figure 5A, B). The measurements revealed a very strong correlation in the placement of the MatP-labeled Ter macrodomain and the Z-ring in Δ*slmA* Δ*min* cells (Figure 5C). Notably, the Z-ring co-localized with the MatP focus in all cases, even including cases when the MatP focus was at the nucleoid periphery close to the cell pole. In wild type cells, correlations were also strongly present although in a few cases (4 out of 166) the Z-ring could be observed to localize at the center of the cell when the MatP locus was close to the cell pole (Figure 5D). These events were also present in the Δ*slmA* single deletion strain but were absent from the Δ*minC* strain (Figure S7) indicating that the Min system reduces correlations between the Z-ring and the Ter macrodomain. The measured co-localization precision between the MatP-labeled Ter foci and Z-ring centers was σ*_X_*_z-*X*MatP_ = 56 nm for Δ*slmA* Δ*min* (Figure 5E) and σ*_X_*_z-*X*MatP_ = 66 nm for wild type cells (Figure 5F). Similar values for σ*_X_*_z-*X*MatP_ also were found for *ΔslmA* and *Δmin* single deletion strains (Figure S7). All the measurements of co-localization precision σ*_X_*_z-*X*MatP_ were close to our resolution limit and thus consistent with the hypothesis that the Ter macrodomain and Z-ring co-localize in *E. coli* unless the Min system prevents such co-localization from happening.

### The Ter region arrives at the cell center before Z-ring formation

**Figure 6:**
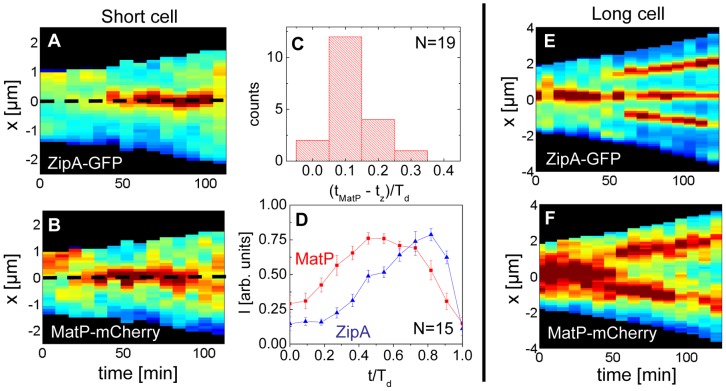
Arrival of the MatP foci and the Z-ring at midcell. (A) Distribution of ZipA-GFP along the cell length as a function of time for a short Δ*slmA* Δ*min* cell (strain WD1). (B) Distribution of MatP-mCherry labeled Ter region for the same cell. In the heat maps blue corresponds to low and red to high intensity. The dashed black line approximately marks midcell. (C) Histogram of time differences between the arrival of MatP (t_MatP_) and ZipA (t_z_) at midcell. The times are expressed in doubling times. (D) Accumulation of ZipA-GFP (blue triangles) and MatP-mCherry (red rectangles) at midcell as a function of time. Each curve represents the average from measurements of 15 cells. Error bars represent standard errors. (E) Distribution of ZipA-GFP and (F) MatP-mCherry in a long Δ*slmA* Δ*min* cell.

Previously, it was argued that the *divisome* anchors the Ter macrodomain to the cell center through a MatP-mediated link in which the divisome related proteins ZapA and ZapB participate [25]. The co-localization data (Figure 5) clearly supports the presence of this link, which we refer to as the Ter linkage. The data also raise the possibility that the Ter macrodomain may be important in positioning and stabilizing the location of the divisome. If the latter hypothesis is correct then there should be some time delay between the arrival of the Ter macrodomain at the cell center and the subsequent formation of the Z-ring. To test this hypothesis we followed the movement of the Ter macrodomain and the Z-ring in Δ*slmA* Δ*min* and wild type cells using MatP-mCherry and ZipA-GFP labels. Similar to an earlier report on wild type cells [25], [28], in Δ*slmA* Δ*min* cells under slow growth conditions the Ter macrodomain moved from the cell pole to the center of the cell at the beginning of the cell cycle (Figure 6 A–B, Figure S8, Movie M1, M2). During this movement, the Ter macrodomain either split into two distinct foci or displaced through the cell as a somewhat diffuse unit. The Ter region of the chromosome remained in the center of the nucleoid for the majority of the cell cycle before splitting into two foci during the late stage of cytokinesis. The Z-ring co-localized with the Ter macrodomain early in the cell cycle when the Ter region was positioned at the cell poles and during the majority of the cell cycle when the Ter region was localized as a single unit at midcell (Figure 6 A–B). However, our measurements showed that during the period in which the Ter macrodomain dislocated from the new pole to the cell center, the ZipA-GFP focus lagged behind the MatP-labeled Ter macrodomain. We measured the lag period to be (0.12±0.07)⋅T_d_ for Δ*slmA* Δ*min* cells (Figure 6C). The doubling time, T_d_, was about 120 min in these growth conditions. In addition to the lag period, the accumulation of the Z-ring proteins and the Ter macrodomain in the center of the cell showed different time-dependent behaviors (Figure 6D). Following the beginning of the cell cycle, the MatP-mCherry labeled Ter macrodomain arrived at the cell center not only with a shorter delay but also accumulated in the center of the cell *on average* more rapidly than the ZipA-GFP marker for the Z-ring (Figure 6D). We observed a similar behavior for wild type cells (Figure S9) although the delay appeared somewhat smaller, (0.02±0.10) ⋅T_d_ (Figure S10).

Time lapse measurements of the Ter macrodomain and the Z-ring in longer (L>6 µm) Δ*slmA* Δ*min* cells indicate why these cells prefer divisions at the ¼ positions from the cell poles (cf. Figure 2A) and preferentially show Z-rings at the new division sites (cf. Figure 4). The measurements showed that a shift from the cell center to ¼ positions occurred when the Ter region moved from the center of the cell to ¼ positions from the cell poles (Figure 6 E–F, Movie M3). This was shortly accompanied by an appearance of the Z-rings in the same locations. In some cases we observed that the Z-ring completely disappeared from the central location, while in other cases, as shown in Figure 6 E–F, the Z-ring also persisted in the cell center and was able to complete division. Observations that the Z-ring follows the movement of the Ter macrodomain in a highly correlated manner for both single and multi-nucleoid Δ*slmA* Δ*min* cells are consistent with the hypothesis that the Ter macrodomain acts as a positional landmark for cell division proteins in these cells.

### Localization of the Z-ring in the absence of the putative Ter linkage

**Figure 7:**
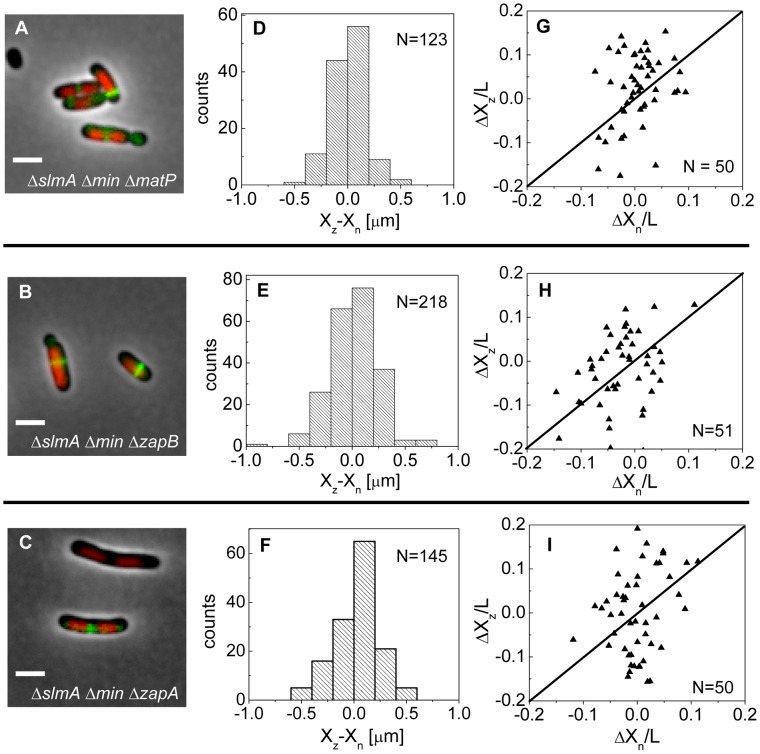
Positioning of the Z-rings relative to the cell and nucleoid centers in triple deletion strains. Composite of DAPI labelled nucleoid (red), ZipA-GFP (green) and phase contrast image in (A) Δ*slmA* Δ*min* Δ*matP*, (B) Δ*slmA* Δ*min* Δ*zapB*, and (C) Δ*slmA* Δ*min* Δ*zapA* cells. Scale bar is 2 µm. (D)–(F) Distribution of distances between the Z-ring center and nucleoid center for Δ*slmA* Δ*min* Δ*matP*, Δ*slmA* Δ*min* Δ*zapB*, and Δ*slmA* Δ*min* Δ*zapA* cells, respectively. (G)–(I) ΔX_z_ vs. ΔX_n_ in Δ*slmA* Δ*min* Δ*matP*, Δ*slmA* Δ*min* Δ*zapB*, and Δ*slmA* Δ*min* Δ*zapA* cells, respectively. Data are from cells with a single compact nucleoid and a central Z-ring. Straight lines correspond to 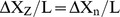.

If the MatP-ZapB-ZapA linkage is involved in the co-localization of the Ter macrodomain and the Z-ring in Δ*slmA* Δ*min* cells, then rendering the linkage dysfunctional by removal of any proteins of the linkage should make the placement of the Z-ring relative to the nucleoid center more random. To verify this prediction we constructed Δ*slmA* Δ*min* Δ*matP*, Δ*slmA* Δ*min* Δ*zapB*, and Δ*slmA* Δ*min* Δ*zapA* triple deletion strains. The triple mutants were imaged using ZipA-GFP as a Z-ring label and DAPI as a stain for nucleoids (Figure 7 A–C). Indeed, the distributions of distances between the central Z-ring and nucleoid centers (Figure 7 D–F) were more than a factor of two wider after deletion of *matP* (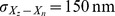; 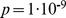), *zapB* (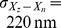; 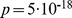) and *zapA* (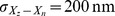; 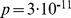) from Δ*slmA* Δ*min* cells (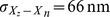). All p-values were calculated using single tailed Ansari-Bradley test. Note that the horizontal axes in Figure 7 D–F spans a distance that is three times larger than in the corresponding graphs for Δ*slmA* Δ*min* and the parental cells (Figure 3 E–F). Wider X_z_−X_n_ distributions for the Δ*slmA* Δ*min* Δ*zapA* and Δ*slmA* Δ*min* Δ*zapB* strains compared to Δ*slmA* Δ*min* Δ*matP* strain are likely caused by irregular Z-ring patterns in the former two strains. ZapA and ZapB have been identified as bundling agents for the FtsZ protofilaments [30], [31]. In the absence of these proteins aberrantly shaped Z-rings can be present at the division site which leads to higher uncertainty in Z-ring positions.

We also observed a significantly higher percentage of polar Z-rings (Figure S11) and polar constrictions after deletion of *matP*, *zapB*, and *zapA* from Δ*slmA* Δ*min* background (Table S3). Note that only a fraction of polar Z-rings leads to polar constrictions. For example, in Δ*slmA* Δ*min* Δ*matP* cells the frequency of polar Z-rings was 69% while the frequency of polar constrictions leading to minicelling divisions was 28%. Although the division planes were positioned much more randomly in triple deletion strains than in the Δ*slmA* Δ*min* strain, the cell length distributions (Figure S12) were not significantly affected except for Δ*slmA* Δ*min* Δ*matP* strain which was longer. Constancy of cell length may indicate that the timing and duration of cell division are not affected by *zapA* and *zapB* deletions but may be affected by *matP* deletion. Considering MatP is also involved in organizing the Ter region of chromosome [28], it is conceivable that its deletion could affect cell length more so than a deletion of ZapA or ZapB. Altogether, these findings show that the Ter linkage strongly affects the accuracy and precision of division plane placement but it appears not to affect significantly the timing of cell division.

Consistent with the role of ZapB and MatP in the Ter linkage, we observed a drastic loss of co-localization between the Z-rings and MatP foci in Δ*slmA* Δ*min* Δ*zapB* cells and in Δ*slmA* Δ*min* cells when the last 20 amino acids in the C-terminus of MatP were replaced by an mCherry fusion (*matPΔC*-mCherry) (Figure S13). The MatP C-terminal domain has been shown to be important for its interaction with ZapB [25]. While our data indicates that the Ter linkage determines the position of the Z-ring, it has been shown that the linkage is required to stabilize the position of the Ter macrodomain [25]. Our data do not contradict this finding. The MatP focus appeared more delocalized relative to the nucleoid center in the absence of the Ter link in the Δ*slmA* Δ*min* Δ*zapB* and Δ*slmA* Δ*min* Δ*matPΔC-mCherry* strains compared to the Δ*slmA* Δ*min* and wild type strains (Figure S14). The Ter linkage thus appears to determine the position of the Z-ring and at the same time stabilize the position of the Ter macrodomain relative to the cell center once the Z-ring has formed.

In cells with compact nucleoids, representative of an early state of chromosome segregation, analysis of Z-ring positions relative to nucleoid-centers revealed essentially no co-localization (Figure 7G–I). The corresponding 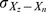 values for Δ*slmA* Δ*min* Δ*matP*, Δ*slmA* Δ*min* Δ*zapB*, and Δ*slmA* Δ*min* Δ*zapA* strains were about a factor of 1.5 larger (230 nm, 344 nm, 280 nm, respectively) than these values for the whole cell population. These differences were statistically significant in both the F-test and in the Ansari-Bradley test. This evidence suggests that the Ter linkage is critical to the specific localization of the Z-ring with the chromosomal terminus at early states of chromosome segregation when the nucleoid morphology is compact. Once the bi-lobed nucleoid morphology emerges in the triple deletion strains, co-localization between the Z-ring and nucleoid center appears, though much more weakly than in the Δ*slmA* Δ*min* and parental strains.

Interestingly, the spatial distributions of those Z-rings that were not located at the poles still displayed a bias towards the cell center (Figure 7 D–F, Figure S11). The locations of constrictions in the triple deletion strains, which we measured from phase contrast images, showed an overall positioning bias towards cell centers as well (Figure S15). However, the corresponding distributions were significantly broader in triple deletion strains than in Δ*slmA* Δ*min* cells. The latter findings indicate that while triple deletion strains lack a mechanism to recognize centers of compact nucleoids, they still have a mechanism that can position Z-rings relative to cell center albeit with significantly lower precision and accuracy than the Δ*slmA* Δ*min* and parental strains.

## Discussion

The Min system and SlmA-mediated nucleoid occlusion are the only two molecular systems responsible for positioning the cytokinetic ring in *E. coli* that have been identified thus far [2]–[5]. Here, we show that *E. coli* without these two known positioning systems is capable of coordinating cell division and chromosome segregation with high fidelity. The majority of Δ*slmA* Δ*min* cells position their division planes accurately relative to nucleoids in slow growth conditions and produce essentially no minicells. In searching for the mechanism responsible for the localization of the Z-ring in these double mutant cells, we found that the Z-rings have a strong tendency to co-localize with the nucleoid centers. Further investigation showed that the nucleoid centers were occupied by the Ter region of the chromosome at the time of Z-ring formation.

The Ter region of the *E. coli* chromosome is organized by MatP proteins [28]. MatP links the Ter macrodomain to the Z-ring through ZapB and ZapA proteins [25]. It was proposed earlier that the Z-ring acts as an anchor for the Ter macrodomain through this linkage [25]. Our time lapse measurements show a broader role of the Ter linkage. These measurements demonstrate that the MatP-decorated macrodomain arrives at the cell center a small fraction of the cell cycle before appreciable assembly of the Z-ring occurs in Δ*slmA* Δ*min* cells. This temporal relationship indicates that the Ter region of the chromosome through the Ter linkage localizes the cell division proteins in the early stage of cytokinesis. It is thus the Ter macrodomain that acts as an ‘anchor’ for cell division proteins during the formation of the divisome. However, the interactions between the Z-ring and the Ter macrodomain appear to stabilize the position of Ter macrodomain later in the cell cycle. During maturation of the divisome, especially when it becomes fixed to the cell wall, the divisome acts as a stabilizing element for the Ter macrodomain, holding it fixed in the cell center [25].

### A positive regulation mechanism for cell division

The Ter linkage facilitates correct placement of the division plane relative to the chromosomes. Severing the linkage in Δ*slmA* Δ*min* cells leads to increased number of unviable minicells and less symmetric division of mother cells. Both outcomes limit the fitness of cells. Unlike the Min system and SlmA-mediated nucleoid occlusion, which are inhibitors of Z-ring formation, the Ter linkage represents a positive regulatory mechanism. The link guides cell division proteins to the location of the future division site and not away from the undesired locations in the cell as do the Min system and SlmA-mediated nucleoid occlusion.

The positive regulation by the Ter linkage is dynamic and it is likely not very strong. Time-lapse measurements show that the Ter linkage temporarily disassembles when the Ter region of the chromosome moves from the cell pole to its center. Also, the Ter region becomes disconnected from the divisome near the end of cytokinesis. The Ter linkage appears thus to provide a dynamic and reconfigurable connection, which biases assembly of cell division proteins towards the Ter region, but does not commit cells to division.

The Ter linkage and the Min system define two independent positioning systems for the divisome. The Min system is capable of positioning the Z-ring without any nucleoid in *E. coli* minicells albeit with somewhat lower precision than in wild type cells [32]. The position defined by the Min system may, however, not always match the position defined by the Ter macrodomain. In these conflicting cases, the Min system has the dominant effect over the Ter linkage. Consistent with this idea, we observed in long cephalexin treated wild-type cells that Z-rings localized only at the cell center rather than at the locations of MatP foci. Also, in the Min^+^ cells we observed no appreciable accumulations of the ZipA-GFP reporter near the cell poles although this location is favored by the Ter linkage at the early stages of the cell cycle.

Unlike the Min system, the effect of SlmA on the Ter linkage was less pronounced. The only observed consequence of deleting *slmA* in our measurements was the decrease in polar Z-rings and minicelling divisions in the Δ*slmA* Δ*min* strain compared to the Δ*minC* strain. We hypothesize that SlmA removal, i.e. removal of the negative regulator, effectively strengthens the positive regulation due to the Ter linkage. The stronger regulation due to the Ter linkage then leads to more abundant Z-rings in the vicinity of the Ter region(s) of the chromosome, which sequester more efficiently the Z-ring related proteins from other regions of the cell including cell poles. As a result, less polar Z-rings and minicelling divisions are present in the Δ*slmA* Δ*minC* than in the Δ*minC* cells. More work is needed to further test this hypothesis as well as to understand the exact mechanism of how SlmA regulates Z-ring assembly.

### Positive regulation mechanisms in other bacteria

Evidence of positive control in localizing cell division proteins has been reported recently for several bacterial species including *Streptomyces* [33], *Myxococcus xanthus* [34] and *Bacillus subtilis* [22], [24]. In *Streptomyces* the positive control appears to be achieved by a combination of SsgA and SsgB proteins [33]. In *M. xanthus*, PomZ is shown to have a similar role [34]. Although these proteins arrive before FtsZ in both organisms, it remains unclear which molecular mechanisms are responsible for their own localization. PomZ appears to localize over the nucleoid although it has not been determined if it is linked to any specific chromosomal region [34]. Positioning of SsgA and SsgB relative to chromosome also is not clear yet.

A positive localization signal, or potentiaton as the authors refer to it, appears to be present also in *B. subtilis* [22]. However, the mechanism seems to be very different in *B. subtlis* in which the positive signal was reported to appear during the assembly of the replichore, i.e. much earlier than in *E. coli*. Moreover, it was observed that “some factor” attracted Z-ring assembly to the oldest division site in *B. subtilis* outgrowing spores that lacked Min and Noc proteins [24]. This is contrary to our observation in *E. coli*, where the Z-ring is biased towards sites between newly segregating nucleoids. Taking that *B. subtilis* is evolutionarily divergent from *E. coli*, differences are expected. It remains to be determined how widespread the Ter linkage is among other bacteria. MatP is conserved in enterobacteria [28], but taking its important functional role, structurally similar assemblies can be present more broadly.

### Additional mechanisms for localization of cell division proteins

Deletion of any of the three proteins involved in the Ter linkage affects the midcell positioning of the Z-ring but does not lead to complete positioning randomness. Accordingly, a mechanism responsible for the localization of cell division proteins must exist in addition to the MatP-ZapB-ZapA mediated Ter linkage in Δ*slmA* Δ*min* cells. The mechanism does not appear to link Z-rings to nucleoid centers at early stages of chromosome segregation when there is no discernable bi-lobed nucleoid structure (compact nucleoids). Interestingly, later in chromosome segregation when a distinct bi-lobed morphology appears, stronger correlations between the Z-rings and nucleoid centers emerge. Two positioning mechanisms that link the nucleoid and divisome have been discussed in the past [35], [36] that can possibly explain such behavior. Both mechanisms rely on the transertional linkages that connect bacterial DNA through transcribed RNA and simultaneously translated membrane proteins to the plasma membrane of the cell [37]. In one hypothesis transertional linkages create local membrane crowding [35] that prevents Z-ring formation in the vicinity of the nucleoid. In another hypothesis, mechanical tension produced by the transertional linkages due to chromosomal segregation acts as a (positive) signal to guide localization of cell division proteins [36]. Further work can prove or disprove these ideas.

In conclusion, we have shown that *E. coli* lacking both the Min system and the nucleoid occlusion factor SlmA are able to localize their division planes at the centers of nucleoids as opposed to the nucleoid free regions in slow growth conditions. In this localization process, the Ter region of the chromosome acts as a landmark for the Z-ring. Removal of the Ter linkage, which involves MatP, ZapB, and ZapA proteins, significantly affects the accuracy and precision with which the Z-ring localizes over the nucleoid. Our data, however, is indicative that yet an unidentified, lower fidelity positioning system remains in *E. coli* Δ*slmA* Δ*min* cells even without the Ter linkage. Despite the lower fidelity, this unidentified positioning system still coordinates Z-ring localization relative to the cell center. Further studies are warranted to identify the molecular origins of this positioning mechanism.

## Materials and Methods

### Strains and growth conditions

All strains used in this study were derivatives of *E. coli* K-12. Descriptions of all strains and plasmids are given in Table S1. All bacteria were grown in M9 minimal medium (Sigma-Aldrich) supplemented with magnesium sulfate and either with 0.5% glucose or 0.3% glycerol. 20 µg/ml kanamycin, 35 µg/ml chloramphenicol, 20 µg/ml ampicillin was used to grow the strains with respective resistance markers. 50 µg/ml ampicillin was used to grow strains carrying pKen1-GFPμ2 plasmids. To grow long, multi-nucleoid cells, all strains were incubated with 20 µg/mL of cephalexin for approximately 2 hours. All bacteria were grown and imaged at 28°C.

### Fluorescent microscopy

A Nikon Ti-E inverted fluorescence microscope with a 100X NA 1.40 oil immersion phase contrast objective was used for imaging the bacteria. Fluorescence was excited by a 200W Hg lamp through an ND4 or ND8 neutral density filter. Chroma 41004, 41001 and 31000v2 filtercubes were used to record mCherry, GFP and DAPI images, respectively. Images were captured by an Andor iXon DU897 camera and recorded using NIS-Elements software.

Cells were imaged on M9 agar pads for still imaging. For time lapse imaging home-made glass bottom dishes were used. Cells were pipetted to #1.5 glass coverslips on the bottom of the dish and covered with about 1 cm thick slab of M9 agar. No antibiotics were used in M9 agar during imaging. Agar was supplemented with IPTG (10–40 µM) for strains with ZipA-GFP constructs. For DAPI labeling cells were incubated in 0.2 µg/ml DAPI for 1/2 hour before spreading cells on the pads.

### Image analysis

Matlab with the Image Analysis Toolbox and DipImage Toolbox (http://www.diplib.org/) were used for image analysis. In addition to Matlab, simpler image processing such as contrast and brightness adjustments were performed using ImageJ software (v1.41o). The procedures for finding volume fraction ratios is described in [6]. The procedure for finding nucleoid centers, centers of MatP foci, and Z-ring positions relative to cell center is given in Text S1.

## Supporting Information

Figure S1 Length distribution of daughter cells soon after division when two daughter cells still adhere to each other by their poles. (A) Wild type strain (BW25113), (B) *ΔminC* strain (JW1165), (C) *ΔslmA ΔminC* double mutant strain (PB194).
(TIF)

Figure S2 Images of DAPI stained nucleoid and ZipA-GFP labelled Z-ring for *ΔslmA ΔminC* double mutant strain TB86 (left column) and parental strain JMBW5 (right column). In the bottom row, the two fluorescent images are overlaid with phase contrast image. The scale bars are 2 µm.
(TIF)

Figure S3 Displacements of Z-rings relative to the cell center, ΔX_z_, for (A) Δ*slmA* Δ*min* (strain TB86 with ZipA-GFP labeled Z-ring), (B) Δ*slmA* Δ*min* (strain TB86Dr120 with FtsZ-GFP labeled Z-ring), and (C) parental strain (strain JMBW5 ZipA-GFP labeled Z-ring). Data are shown only for cells with a single nucleoid.
(TIF)

Figure S4 Displacements of Z-rings relative to the cell center, ΔX_z_, as a function of nucleoid displacement, ΔX_n_ for Δ*slmA* Δ*min* cells with FtsZ-GFP label (strain TB86 λDR120). Both displacements are normalized by cell length L. All analysis is pertinent to cells with a single nucleoid. (A) Solid rectangles correspond to central Z-rings and open rectangles for polar rings. (B) The same as (A) but for cells with central Z-rings over compact nucleoids that do not show an apparent dip in their chromosomal distribution. (C) Distribution of distances between the Z-ring center and nucleoid center. Data are collected from cells that have a central Z-ring.
(TIF)

Figure S5Localization of ZipA-GFP labeled Z-rings relative to cell center and the center of nucleoids for Δ*minC* (top row) and Δ*slmA* (bottom row) single deletion strains. (A, B) ΔX_z_ vs. ΔX_n_ scaled by cell length L. Solid rectangles mark central and open rectangles mark polar Z-rings. The solid line corresponds to 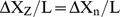 Data are shown only for cells with a single nucleoid. (C, D) Distribution of distances between the Z-ring center and nucleoid center. Only data for central Z-rings are shown. (E, F) ΔX_z_ vs. ΔX_n_ for cells that show a Z-ring over a compact nucleoid.(TIF)

Figure S6 Positioning of Z-rings relative to nucleoids in Δ*slmA* Δ*min* and Δ*slmA* and Δ*minC* single deletion strains after 20 µg/ml cephalexin treatment. (A, D, G) Composite images of cells after cephalexin treatment. ZipA-GFP (green), DAPI stained nucleoid (red), and phase contrast images (grey) have been overlaid. Scale bar is 2 µm. (B, E, H) Nucleoid and ZipA-GFP density distributions along the long axis of the cell for the cell shown in the adjacent left panel. The positions marked by “N” correspond to the new division sites at the centers of the nucleoids and the position marked by “O” to old division site between fully segregated nucleoids. (C, F, I) Frequency of Z-rings in the double mutant cells at the new and old replication sites. Only cells that have two or more distinct nucleoids have been analyzed.
(TIF)

Figure S7Positioning of the Z-ring relative to the MatP-labeled Ter macrodomain Δ*slmA* and Δ*minC* in single deletion cells. (A, D) A composite of ZipA-GFP (green), MatP-mCherry (red), and phase contrast image (grey). Scale bar is 2 µm. (B, E) Location of ZipA-GFP labeled Z-ring (ΔX_z_) vs location of MatP-mCherry focus (ΔX_MatP_). Both locations are referenced relative to the cell center. The straight line represents 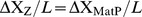. (C, F) Distribution of distances between the Z-ring and the MatP focus along the cell length. In *ΔminC* strain the outliers beyond ±0.3 µm have been left out.(TIF)

Figure S8 Displacement of the Z-ring and MatP-labeled Ter macrodomain for two Δ*slmA* Δ*min* cells (strain WD1). The Z-ring is labeled using a ZipA-GFP construct and Ter macrodomain by a MatP-mCherry construct. (A, B) ZipA-GFP fluorescence intensity along the long axes of the cell (x) as a function of time (t). (C, D) The same for MatP-mCherry intensity. In the heat maps, blue corresponds to low and red to high intensity. The time interval covers one full cell cycle. (E, F) Intensity of ZipA-GFP (blue trace with filled circles) and MatP-mCherry (red trace with open triangles) in the cell center (x = 0 µm) as function of time.
(TIF)

Figure S9 Displacement of the Z-ring and MatP-labeled Ter macrodomain for two wild type cells (strain WD2). The Z-ring is labeled using a ZipA-GFP construct and Ter macrodomain by a MatP-mCherry construct. (A, B) ZipA-GFP fluorescence intensity along the long axes of the cell (x) as a function of time (t). (C, D) The same for MatP-mCherry intensity. In the heat maps, blue corresponds to low and red to high intensity. The time interval covers one full cell cycle. (E, F) Intensity of ZipA-GFP (blue trace with filled circles) and MatP-mCherry (red trace with open triangles) in the cell center (x = 0 µm) as function of time.
(TIF)

Figure S10 Arrival times of MatP and ZipA to the cell center in wild type strain WD2 with MatP-mCherry and ZipA-GFP labels. (A) Histogram of time differences between arrival times of MatP and ZipA. The times are expressed in doubling times. The average and standard deviation of the distribution are (0.02±0.10)T_d_. (B) Accumulation of ZipA-GFP (red rectangles) and MatP-mCherry (blue triangles) in the center of the cell as a function of time. Each curve is average of measurements in 11 cells. Error bars represent standard errors.
(TIF)

Figure S11Displacements of Z-rings relative to the cell center, ΔX_z_, as a function of nucleoid displacement, ΔX_n_ for Δ*slmA* Δ*min* Δ*matP* (A), Δ*slmA* Δ*min* Δ*zapB* (B), and Δ*slmA* Δ*min* Δ*zapA* cells (C). All displacements are normalized by cell length *L*. Solid rectangles mark central and open rectangles polar Z-rings. The solid line corresponds to 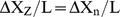. Data are shown only for cells with a single nucleoid.(TIF)

Figure S12 Distance distribution of visible constrictions in mother cells based on phase contrast images. Distances shown are measured from each of the two cell poles. Note that *x*_constriction_ is somewhat smaller than the length of newborn daughter cells (*L*_daughter_) which are shown in .
(TIF)

Figure S13Positioning of the Z-ring relative to the MatP-labeled Ter macrodomain in Δ*slmA* Δ*min* Δ*zapB* (top row) and Δ*slmA* Δ*min matP*Δ*C* (bottom row) strains. (A, B) A composite of ZipA-GFP (green), MatP-mCherry (red), and phase contrast image (grey). Scale bar is 2 µm. (C, D) Location of ZipA-GFP labeled Z-ring (ΔX_z_) vs location of MatP-mCherry focus (ΔX_MatP_). Both locations are referenced relative to the cell center. The straight line represents 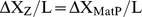. (E, F) Distribution of distances between the Z-ring and the MatP focus along the cell length.(TIF)

Figure S14
**Left column:** Displacements of MatP-focus relative to cell center, ΔX_MatP_, as a function of nucleoid displacement from cell center, ΔX_n_. All displacements are normalized by cell length *L*. The solid line corresponds to 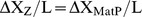. Data are shown only for cells with a single nucleoid. The large scatter in ΔX_MatP_/L values in all strains is related to the movement of the Ter macrodomain from the nucleoid periphery to the center of the nucleoid early in the cell cycle. In the Δ*slmA* Δ*min matPΔC* strain, the movement of Ter macrodomain occurs before cell division. Consequently, in single nucleoid cells no MatP foci appear at the nucleoid periphery. **Right column:** Distance between nucleoid center and center of MatP focus. Each histogram is compiled from the data on the left column but retaining only these data where ΔX_MatP_ is less than 0.25 µm from the nucleoid center. This selection eliminates spread caused by the cell cycle dependent movement of MatP focus from nucleoid periphery to nucleoid center.(TIF)

Figure S15 Placement of constrictions in (A) Δ*slmA* Δ*minC* (strain PB194), (B) Δ*slmA* Δ*minC* Δ*zapA* (strain PB300), (C) Δ*slmA* Δ*minC* Δ*zapB* cells (strain PB299), and (D) Δ*slmA* Δ*minC* Δ*matP* cells (strain PB301). Each constriction is measured relative to two different poles and contributes two values to a given histogram that are located symmetrically to 0.5. Note that the placement of constrictions, which are determined from phase contrast images, differ slightly from the final volume fractions (as shown in Figure 1 in the main text). Constrictions appear closer to mid-cell than the division ratios. For example, constrictions that lead to partitioning of 1 nucleoid to one and 3 nucleoids to another daughter cell are centered at 0.29 in this plot instead of 0.25.
(TIF)

Table S1 List of the strains and plasmids used in experiments.
(DOC)

Table S2Statistics describing co-localization of the Z-ring and the nucleoid center in different strains. *R* ^2^ is a dimensionless goodness of fit parameter for a model 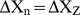. Note that for perfect co-localization of nucleoid and the Z-ring centers, *R^2^* approaches a value of one. *R^2^* can also be negative; for 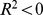 there is no meaningful evidence of co-localization in the data. For cells with compact nucleoid morphology the percentage in parenthesis shows the frequency of those cells in the total population. Here, the total population accounts for all cells that have a single nucleoid and a single Z-ring.(DOC)

Table S3 Frequency of polar Z-rings and minicelling divisions.
(DOC)

Movie S1 Displacement of the Z-ring and the Ter macrodomain in Δ*slmA* Δ*min* double mutant *E. coli* (strain WD1) during one cell cycle. Z-ring fluorescence is followed using a ZipA-GFP construct (green), while the chromosomal terminus is labeled with MatP-mCherry (red). A phase contrast image (gray) is overlaid to visualize the cell contour. The scale bar is 2 µm.
(AVI)

Movie S2 Displacement of the Z-ring and the Ter macrodomain in Δ*slmA* Δ*min* double mutant *E. coli* during one cell cycle. Z-ring fluorescence is followed using a ZipA-GFP construct (green), while the chromosomal terminus is labeled with MatP-mCherry (red). A phase contrast image (gray) is overlaid to visualize the cell contour. The scale bar is 2 µm.
(AVI)

Movie S3 Displacement of the Z-ring and the Ter macrodomain in a long Δ*slmA* Δ*min* double mutant *E. coli* cell. Z-ring fluorescence is followed using a ZipA-GFP construct (green), while the chromosomal terminus is labeled with MatP-mCherry (red). A phase contrast image (gray) is overlaid to visualize the cell contour. The scale bar is 2 µm.
(AVI)

Text S1 Description of image analysis algorithms to find positions of Z-rings, MatP, and the nucleoid centers relative to the cell center.
(DOC)

## References

[1] Bi E, Lutkenhaus J (1991) FtsZ ring structure associated with division in *Escherichia coli* . Nature 354: 161–164. 1944597 10.1038/354161a0

[2] Margolin W (2005) FtsZ and the division of prokaryotic cells and organelles. Nat Rev Mol Cell Biol 6: 862–871. 16227976 10.1038/nrm1745 PMC4757588

[3] Lutkenhaus J (2007) Assembly dynamics of the bacterial MinCDE system and spatial regulation of the Z ring. Annu Rev Biochem 76: 539–562. 17328675 10.1146/annurev.biochem.75.103004.142652

[4] Adams DW, Errington J (2009) Bacterial cell division: assembly, maintenance and disassembly of the Z ring. Nat Rev Microbiol 7: 642–653. 19680248 10.1038/nrmicro2198

[5] de Boer PAJ (2010) Advances in understanding *E. coli* cell fission. Curr Opin Microbiol 13: 730–737. 20943430 10.1016/j.mib.2010.09.015 PMC2994968

[6] Männik J, Wu F, Hol FJH, Bissichia P, Sherratt DJ, et al (2012) Robustness and accuracy of cell division in *Escherichia coli* in diverse cell shapes. Proc Natl Acad Sci U S A 109: 6957–6962. 22509007 10.1073/pnas.1120854109 PMC3345019

[7] Yu XC, Margolin W (1999) FtsZ ring clusters in min and partition mutants: role of both the Min system and the nucleoid in regulating FtsZ ring localization. Mol Microbiol 32: 315–326. 10231488 10.1046/j.1365-2958.1999.01351.x

[8] Den Blaauwen T, Buddelmeijer N, Aarsman MEG, Hameete CM, Nanninga N (1999) Timing of FtsZ assembly in *Escherichia coli* . J Bacteriol 181: 5167–5175. 10464184 10.1128/jb.181.17.5167-5175.1999 PMC94019

[9] Guberman JM, Fay A, Dworkin J, Wingreen NS, Gitai Z (2008) PSICIC: Noise and asymmetry in bacterial division revealed by computational image analysis at sub-pixel resolution. PLoS Comput Biol 4: e1000233. 19043544 10.1371/journal.pcbi.1000233 PMC2581597

[10] Shapiro L, McAdams HH, Losick R (2009) Why and how bacteria localize proteins. Science 326: 1225–1228. 19965466 10.1126/science.1175685 PMC7531253

[11] Raskin DM, de Boer PAJ (1999) Rapid pole-to-pole oscillation of a protein required for directing division to the middle of *Escherichia coli* . Proc Natl Acad Sci U S A 96: 4971–4976. 10220403 10.1073/pnas.96.9.4971 PMC21801

[12] Shen B, Lutkenhaus J (2010) Examination of the interaction between FtsZ and MinCN in *E. coli* suggests how MinC disrupts Z rings. Mol Microbiol 75: 1285–1298. 20132438 10.1111/j.1365-2958.2010.07055.x

[13] Huang KC, Meir Y, Wingreen NS (2003) Dynamic structures in *Escherichia coli*: Spontaneous formation of MinE rings and MinD polar zones. Proc Natl Acad Sci U S A 100: 12724–12728. 14569005 10.1073/pnas.2135445100 PMC240685

[14] Mulder E, Woldringh CL (1989) Actively replicating mucleoids influence positioning of division sites in *Escherichia coli* filaments forming cells lacking DNA. J Bacteriol 171: 4303–4314. 2666394 10.1128/jb.171.8.4303-4314.1989 PMC210205

[15] Woldringh CL, Mulder E, Huls PG, Vischer N (1991) Toporegulation of bacterial division according to the nucleoid occlusion model. Res Microbiol 142: 309–320. 1925029 10.1016/0923-2508(91)90046-d

[16] Bernhardt TG, de Boer PAJ (2005) SlmA, a nucleoid-associated, FtsZ binding protein required for blocking septal ring assembly over chromosomes in *E. coli* . Mol Cell 18: 555–564. 15916962 10.1016/j.molcel.2005.04.012 PMC4428309

[17] Wu LJ, Errington J (2004) Coordination of cell division and chromosome segregation by a nucleoid occlusion protein in *Bacillus subtillis* . Cell 117: 915–925. 15210112 10.1016/j.cell.2004.06.002

[18] Tonthat NK, Arold ST, Pickering BF, Van Dyke MW, Liang SD, et al (2011) Molecular mechanism by which the nucleoid occlusion factor, SlmA, keeps cytokinesis in check. EMBO J 30: 154–164. 21113127 10.1038/emboj.2010.288 PMC3020112

[19] Cho HB, McManus HR, Dove SL, Bernhardt TG (2011) Nucleoid occlusion factor SlmA is a DNA-activated FtsZ polymerization antagonist. Proc Natl Acad Sci U S A 108: 3773–3778. 21321206 10.1073/pnas.1018674108 PMC3048121

[20] Cho HB, Bernhardt TG (2013) Identification of the SlmA active site responsible for blocking bacterial cytokinetic ring assembly over the chromosome. PLoS Genet 9: e1003304. 23459366 10.1371/journal.pgen.1003304 PMC3573117

[21] Wu LJ, Ishikawa S, Kawai Y, Oshima T, Ogasawara N, et al (2009) Noc protein binds to specific DNA sequences to coordinate cell division with chromosome segregation. EMBO J 28: 1940–1952. 19494834 10.1038/emboj.2009.144 PMC2711181

[22] Moriya S, Rashid RA, Rodrigues CDA, Harry EJ (2010) Influence of the nucleoid and the early stages of DNA replication on positioning the division site in *Bacillus subtilis* . Mol Microbiol 76: 634–647. 20199598 10.1111/j.1365-2958.2010.07102.x

[23] Bernard R, Marquis KA, Rudner DZ (2010) Nucleoid occlusion prevents cell division during replication fork arrest in *Bacillus subtilis* . Mol Microbiol 78: 866–882. 20807205 10.1111/j.1365-2958.2010.07369.x PMC2978284

[24] Rodrigues CDA, Harry EJ (2012) The Min system and nucleoid occlusion are not required for identifying the division site in *Bacillus subtilis* but ensure its efficient utilization. PLoS Genet 8: e1002561. 22457634 10.1371/journal.pgen.1002561 PMC3310732

[25] Espeli O, Borne R, Dupaigne P, Thiel A, Gigant E, et al (2012) A MatP-divisome interaction coordinates chromosome segregation with cell division in *E. coli* . EMBO J 31: 3198–3211. 22580828 10.1038/emboj.2012.128 PMC3400007

[26] Wang XD, Possoz C, Sherratt DJ (2005) Dancing around the divisome: asymmetric chromosome segregation in Escherichia coli. Genes Dev 19: 2367–2377. 16204186 10.1101/gad.345305 PMC1240045

[27] Fisher JK, Bourniquel A, Witz G, Weiner B, Prentiss M, et al (2013) Four-dimensional imaging of *E. coli* nucleoid organization and dynamics in living cells. Cell 153: 882–895. 23623305 10.1016/j.cell.2013.04.006 PMC3670778

[28] Mercier R, Petit M-A, Schbath S, Robin S, El Karoui M, et al (2008) The MatP/matS site-specific system organizes the terminus region of the *E. coli* chromosome into a macrodomain. Cell 135: 475–485. 18984159 10.1016/j.cell.2008.08.031

[29] Niki H, Yamaichi Y, Hiraga S (2000) Dynamic organization of chromosomal DNA in Escherichia coli. Genes Dev 14: 212–223. 10652275 PMC316355

[30] Galli E, Gerdes K (2010) Spatial resolution of two bacterial cell division proteins: ZapA recruits ZapB to the inner face of the Z-ring. Mol Microbiol 76: 1514–1526. 20487275 10.1111/j.1365-2958.2010.07183.x

[31] Buss J, Coltharp C, Huang T, Pohlmeyer C, Wang S-C, et al (2013) In vivo organization of the FtsZ-ring by ZapA and ZapB revealed by quantitative super-resolution microscopy. Mol Microbiol 89: 1099–1120. 23859153 10.1111/mmi.12331 PMC3894617

[32] Sun Q, Yu XC, Margolin W (1998) Assembly of the FtsZ ring at the central division site in the absence of the chromosome. Mol Microbiol 29: 491–503. 9720867 10.1046/j.1365-2958.1998.00942.x

[33] Willemse J, Borst JW, de Waal E, Bisseling T, van Wezel GP (2011) Positive control of cell division: FtsZ is recruited by SsgB during sporulation of *Streptomyces* . Genes Dev 25: 89–99. 21205868 10.1101/gad.600211 PMC3012939

[34] Treuner-Lange A, Aguiluz K, van der Does C, Gomez-Santos N, Harms A, et al (2013) PomZ, a ParA-like protein, regulates Z-ring formation and cell division in *Myxococcus xanthus* . Mol Microbiol 87: 235–253. 23145985 10.1111/mmi.12094

[35] Zaritsky A, Woldringh CL (2003) Localizing cell division in spherical *Escherichia coli* by nucleoid occlusion. FEMS Microbiol Lett 226: 209–214. 14553913 10.1016/S0378-1097(03)00580-9

[36] Rabinovitch A, Zaritsky A, Feingold M (2003) DNA-membrane interactions can localize bacterial cell center. Journal of Theoretical Biology 225: 493–496. 14615208 10.1016/s0022-5193(03)00292-3

[37] Norris V (1995) Hypothesis - chromosome separation in *Escherichia coli* involves autocatalytic gene expression, transertion and membrane-domain formation. Mol Microbiol 16: 1051–1057. 8577241 10.1111/j.1365-2958.1995.tb02330.x

