# Supplementary figures and images for: Evidence for Divisome Localization Mechanisms Independent of the Min System and SlmA in Escherichia coli
Source: PLoS Genet. 2014 Aug 7;10(8):e1004504. doi: 10.1371/journal.pgen.1004504 (PMC4125044; doi:10.1371/journal.pgen.1004504)

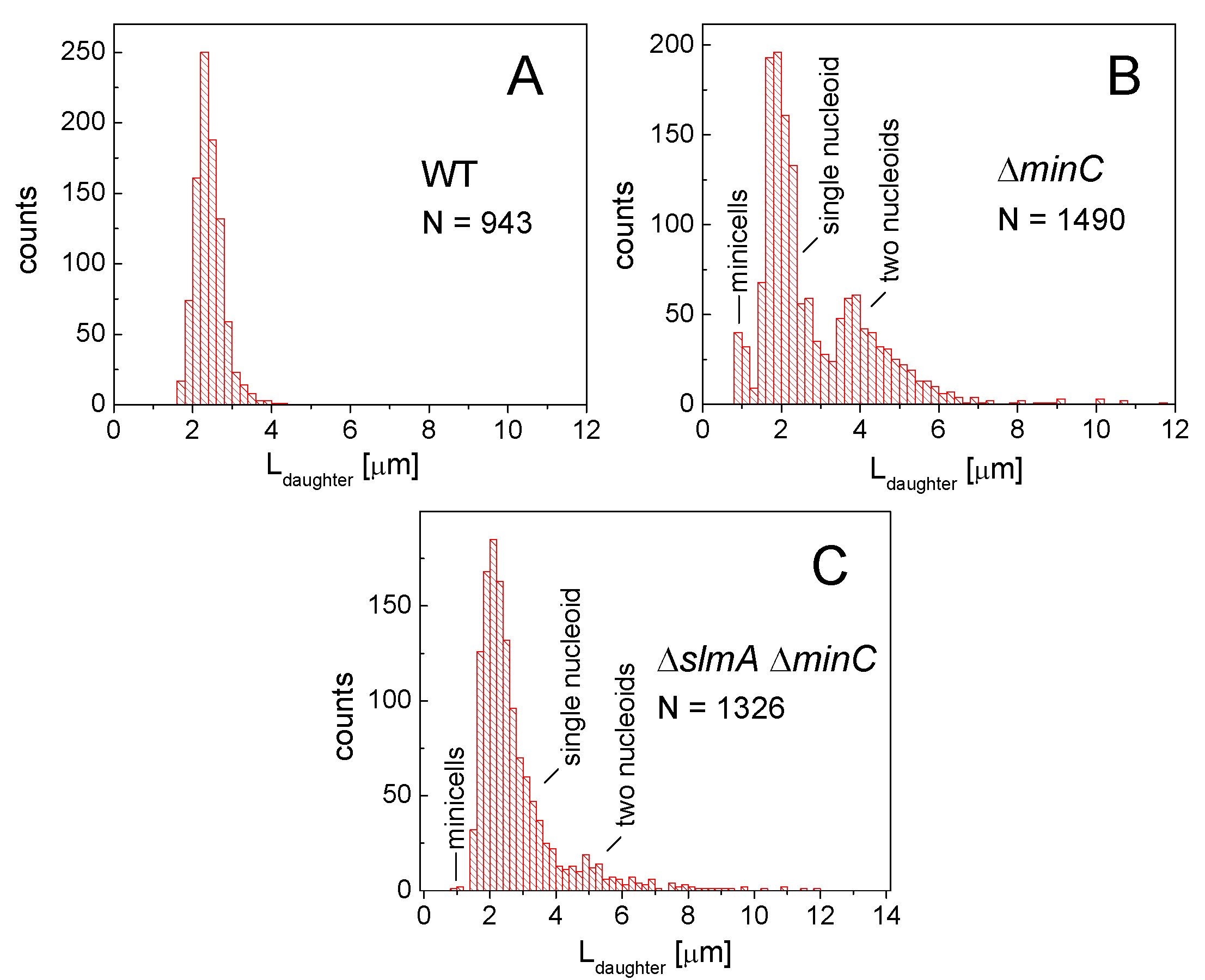

Supplement: Figure S1 — Length distribution of daughter cells soon after division when two daughter cells still adhere to each other by their poles. (A) Wild type strain (BW25113), (B) ΔminC strain (JW1165), (C) ΔslmA ΔminC double mutant strain (PB194). (TIF) [file pgen.1004504.s001.tif]

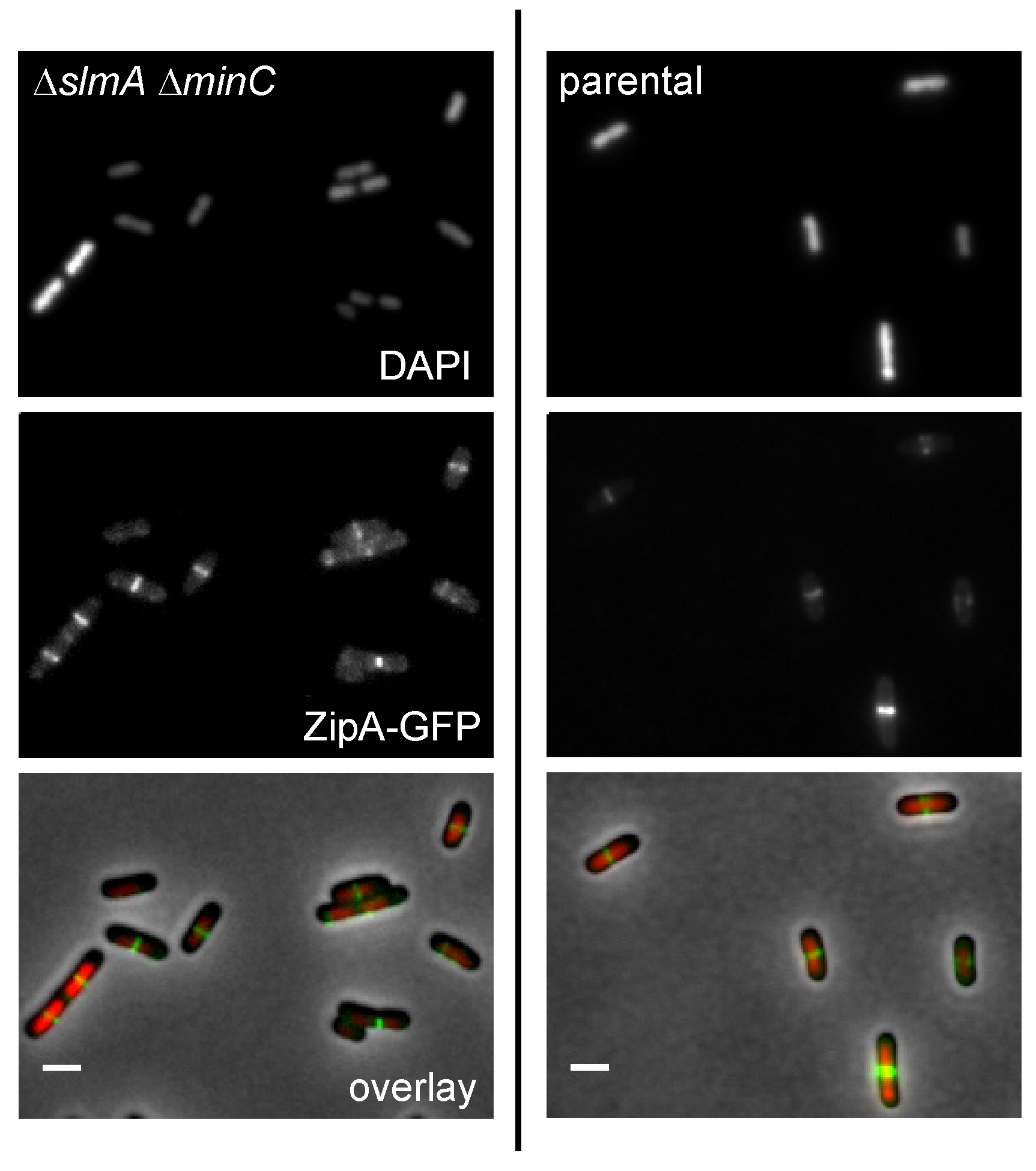

Supplement: Figure S2 — Images of DAPI stained nucleoid and ZipA-GFP labelled Z-ring for ΔslmA ΔminC double mutant strain TB86 (left column) and parental strain JMBW5 (right column). In the bottom row, the two fluorescent images are overlaid with phase contrast image. The scale bars are 2 µm. (TIF) [file pgen.1004504.s002.tif]

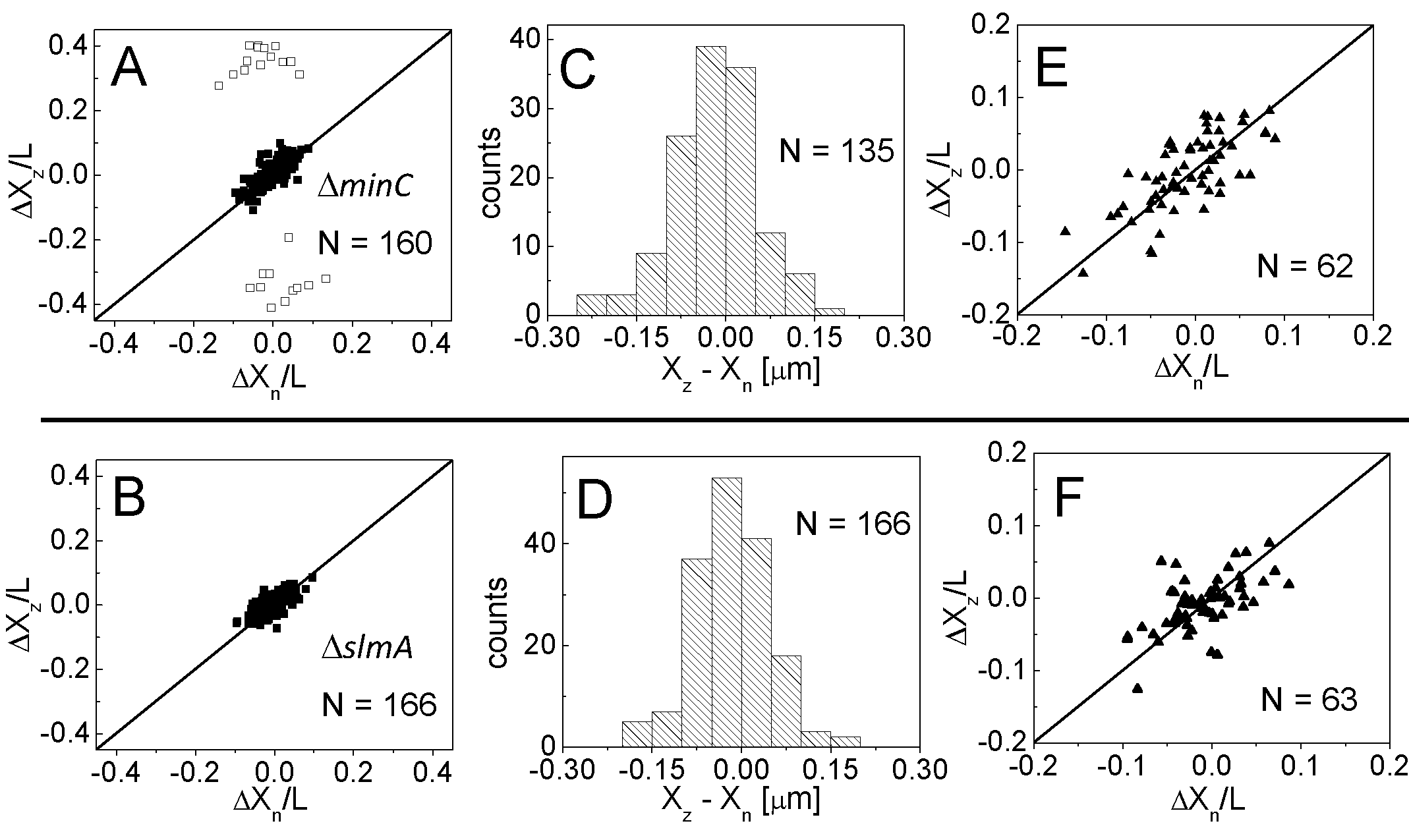

Supplement: Figure S5 — Localization of ZipA-GFP labeled Z-rings relative to cell center and the center of nucleoids for ΔminC (top row) and ΔslmA (bottom row) single deletion strains. (A, B) ΔXz vs. ΔXn scaled by cell length L. Solid rectangles mark central and open rectangles mark polar Z-rings. The solid line corresponds to Data are shown only for cells with a single nucleoid. (C, D) Distribution of distances between the Z-ring center and nucleoid center. Only data for central Z-rings are shown. (E, F) ΔXz vs. ΔXn for cells that show a Z-ring over a compact nucleoid. (TIF) [file pgen.1004504.s005.tif]

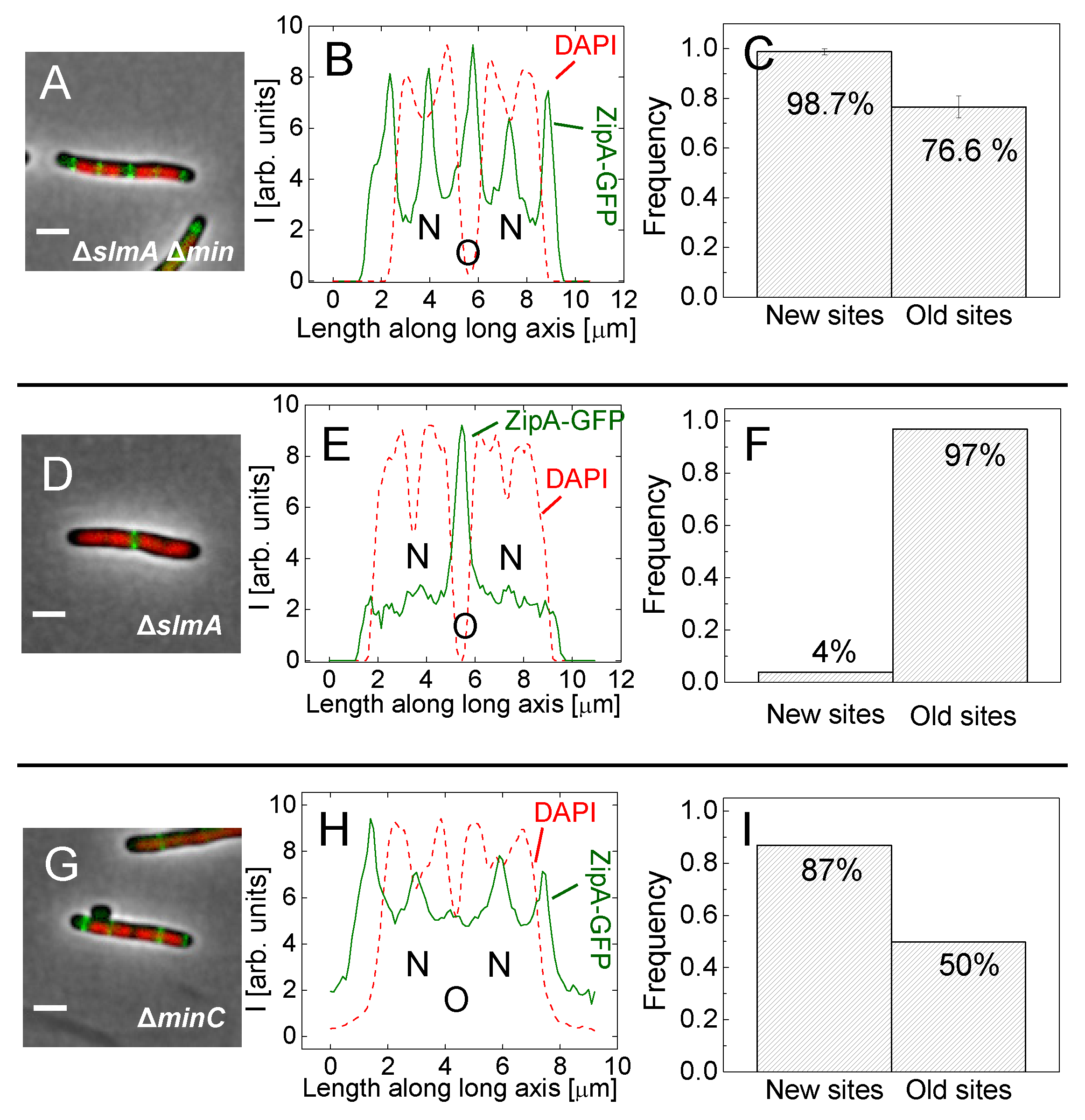

Supplement: Figure S6 — Positioning of Z-rings relative to nucleoids in ΔslmA Δmin and ΔslmA and ΔminC single deletion strains after 20 µg/ml cephalexin treatment. (A, D, G) Composite images of cells after cephalexin treatment. ZipA-GFP (green), DAPI stained nucleoid (red), and phase contrast images (grey) have been overlaid. Scale bar is 2 µm. (B, E, H) Nucleoid and ZipA-GFP density distributions along the long axis of the cell for the cell shown in the adjacent left panel. The positions marked by “N” correspond to the new division sites at the centers of the nucleoids and the position marked by “O” to old division site between fully segregated nucleoids. (C, F, I) Frequency of Z-rings in the double mutant cells at the new and old replication sites. Only cells that have two or more distinct nucleoids have been analyzed. (TIF) [file pgen.1004504.s006.tif]

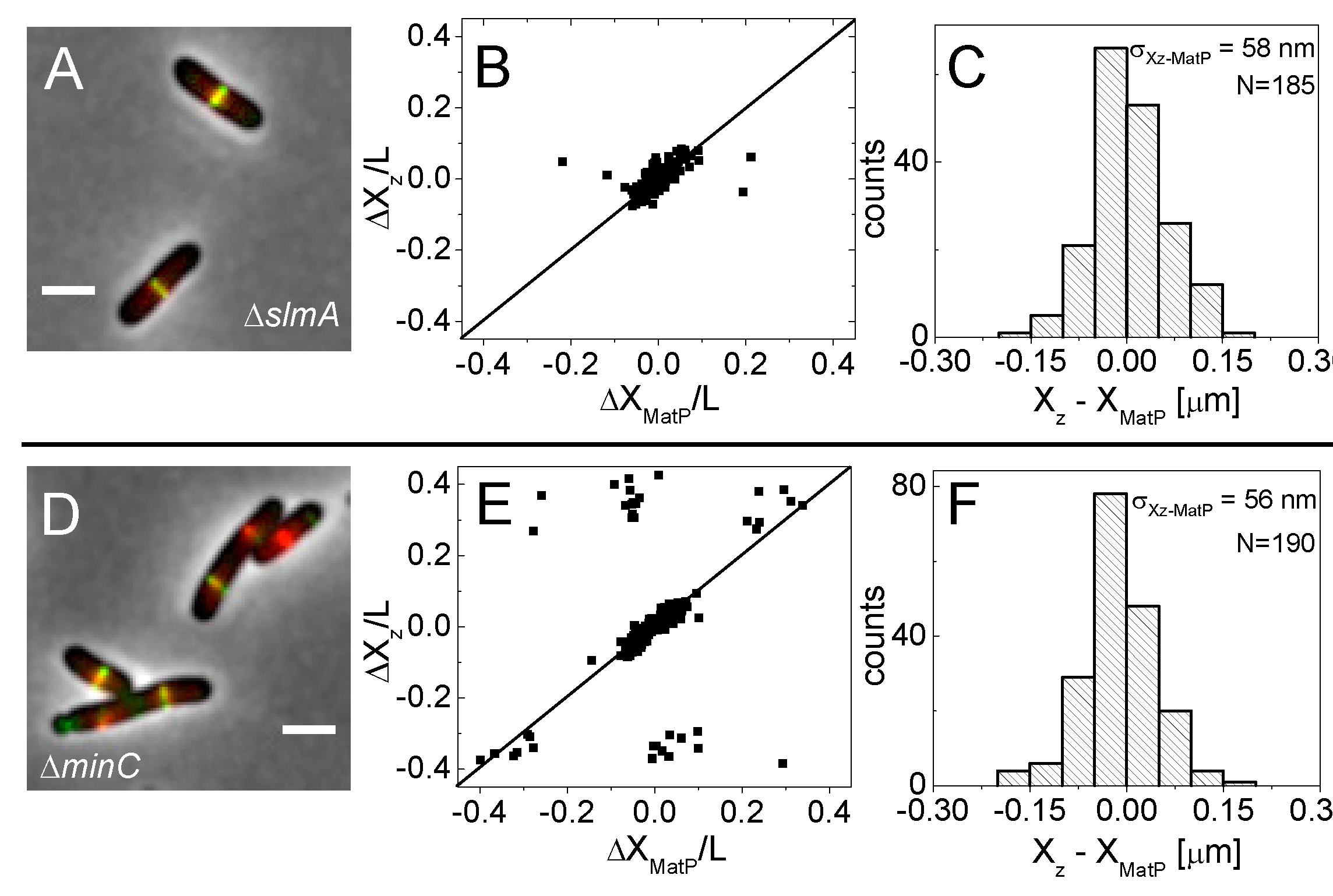

Supplement: Figure S7 — Positioning of the Z-ring relative to the MatP-labeled Ter macrodomain ΔslmA and ΔminC in single deletion cells. (A, D) A composite of ZipA-GFP (green), MatP-mCherry (red), and phase contrast image (grey). Scale bar is 2 µm. (B, E) Location of ZipA-GFP labeled Z-ring (ΔXz) vs location of MatP-mCherry focus (ΔXMatP). Both locations are referenced relative to the cell center. The straight line represents . (C, F) Distribution of distances between the Z-ring and the MatP focus along the cell length. In ΔminC strain the outliers beyond ±0.3 µm have been left out. (TIF) [file pgen.1004504.s007.tif]

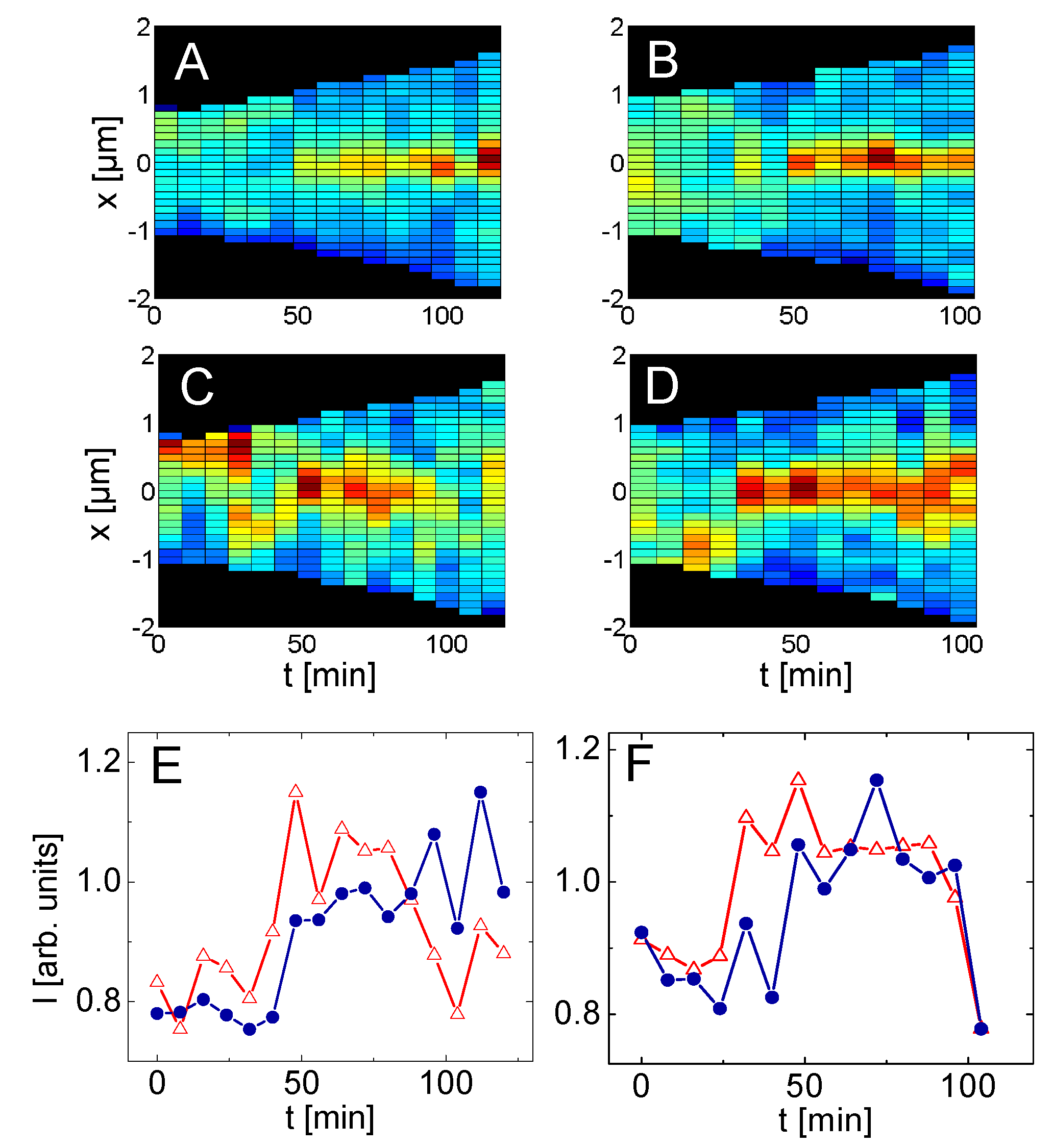

Supplement: Figure S8 — Displacement of the Z-ring and MatP-labeled Ter macrodomain for two ΔslmA Δmin cells (strain WD1). The Z-ring is labeled using a ZipA-GFP construct and Ter macrodomain by a MatP-mCherry construct. (A, B) ZipA-GFP fluorescence intensity along the long axes of the cell (x) as a function of time (t). (C, D) The same for MatP-mCherry intensity. In the heat maps, blue corresponds to low and red to high intensity. The time interval covers one full cell cycle. (E, F) Intensity of ZipA-GFP (blue trace with filled circles) and MatP-mCherry (red trace with open triangles) in the cell center (x = 0 µm) as function of time. (TIF) [file pgen.1004504.s008.tif]

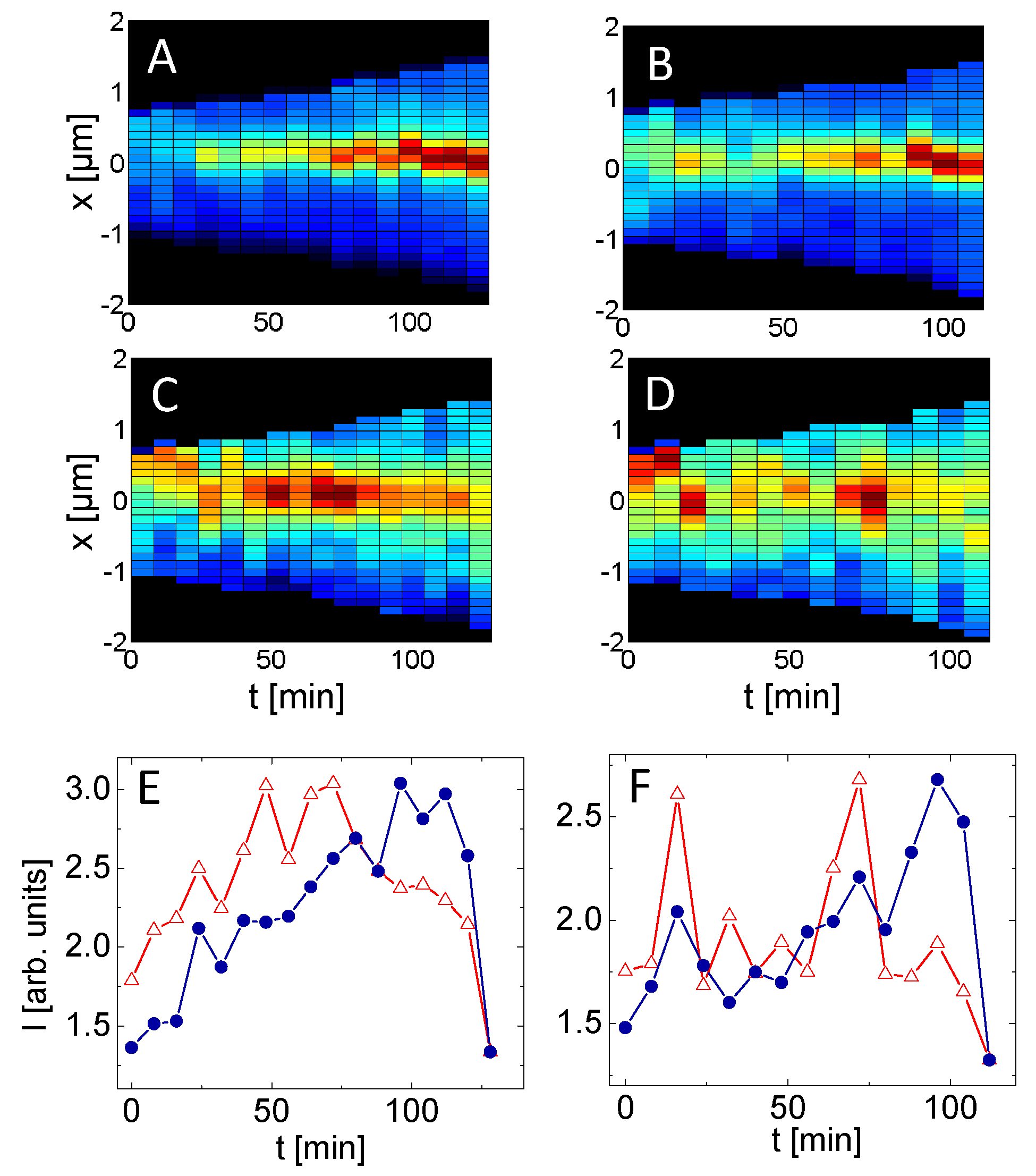

Supplement: Figure S9 — Displacement of the Z-ring and MatP-labeled Ter macrodomain for two wild type cells (strain WD2). The Z-ring is labeled using a ZipA-GFP construct and Ter macrodomain by a MatP-mCherry construct. (A, B) ZipA-GFP fluorescence intensity along the long axes of the cell (x) as a function of time (t). (C, D) The same for MatP-mCherry intensity. In the heat maps, blue corresponds to low and red to high intensity. The time interval covers one full cell cycle. (E, F) Intensity of ZipA-GFP (blue trace with filled circles) and MatP-mCherry (red trace with open triangles) in the cell center (x = 0 µm) as function of time. (TIF) [file pgen.1004504.s009.tif]

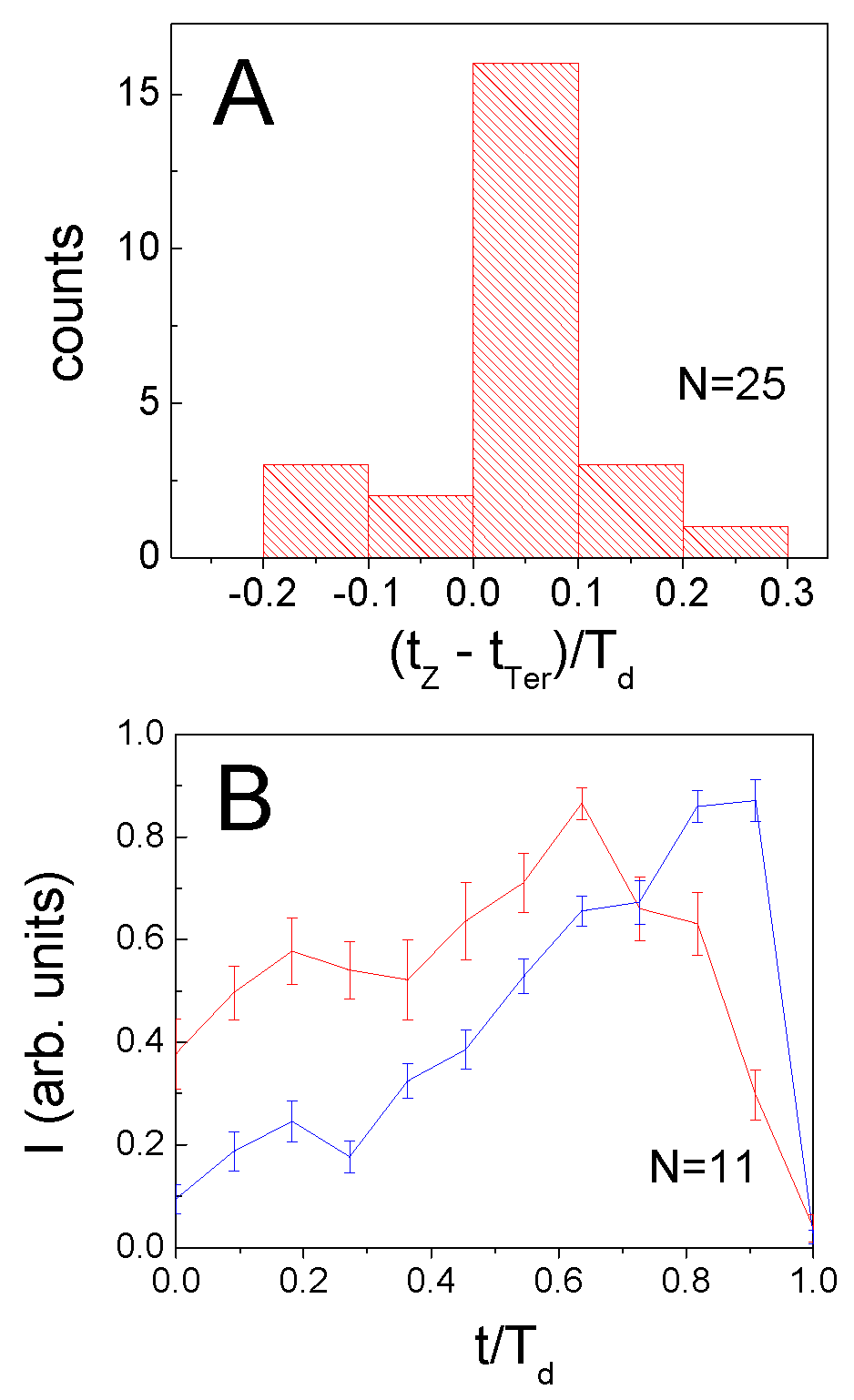

Supplement: Figure S10 — Arrival times of MatP and ZipA to the cell center in wild type strain WD2 with MatP-mCherry and ZipA-GFP labels. (A) Histogram of time differences between arrival times of MatP and ZipA. The times are expressed in doubling times. The average and standard deviation of the distribution are (0.02±0.10)Td. (B) Accumulation of ZipA-GFP (red rectangles) and MatP-mCherry (blue triangles) in the center of the cell as a function of time. Each curve is average of measurements in 11 cells. Error bars represent standard errors. (TIF) [file pgen.1004504.s010.tif]

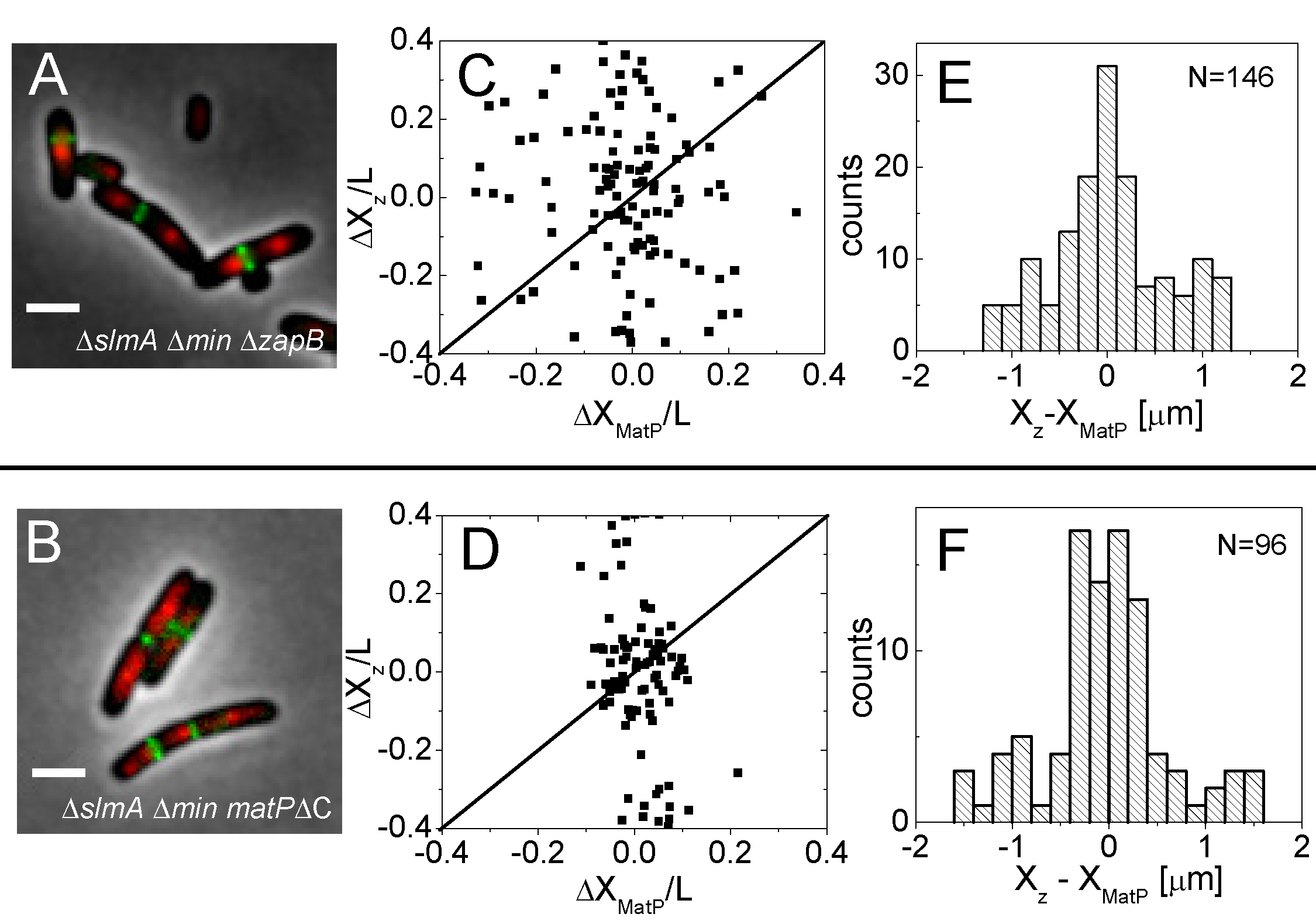

Supplement: Figure S13 — Positioning of the Z-ring relative to the MatP-labeled Ter macrodomain in ΔslmA Δmin ΔzapB (top row) and ΔslmA Δmin matPΔC (bottom row) strains. (A, B) A composite of ZipA-GFP (green), MatP-mCherry (red), and phase contrast image (grey). Scale bar is 2 µm. (C, D) Location of ZipA-GFP labeled Z-ring (ΔXz) vs location of MatP-mCherry focus (ΔXMatP). Both locations are referenced relative to the cell center. The straight line represents . (E, F) Distribution of distances between the Z-ring and the MatP focus along the cell length. (TIF) [file pgen.1004504.s013.tif]

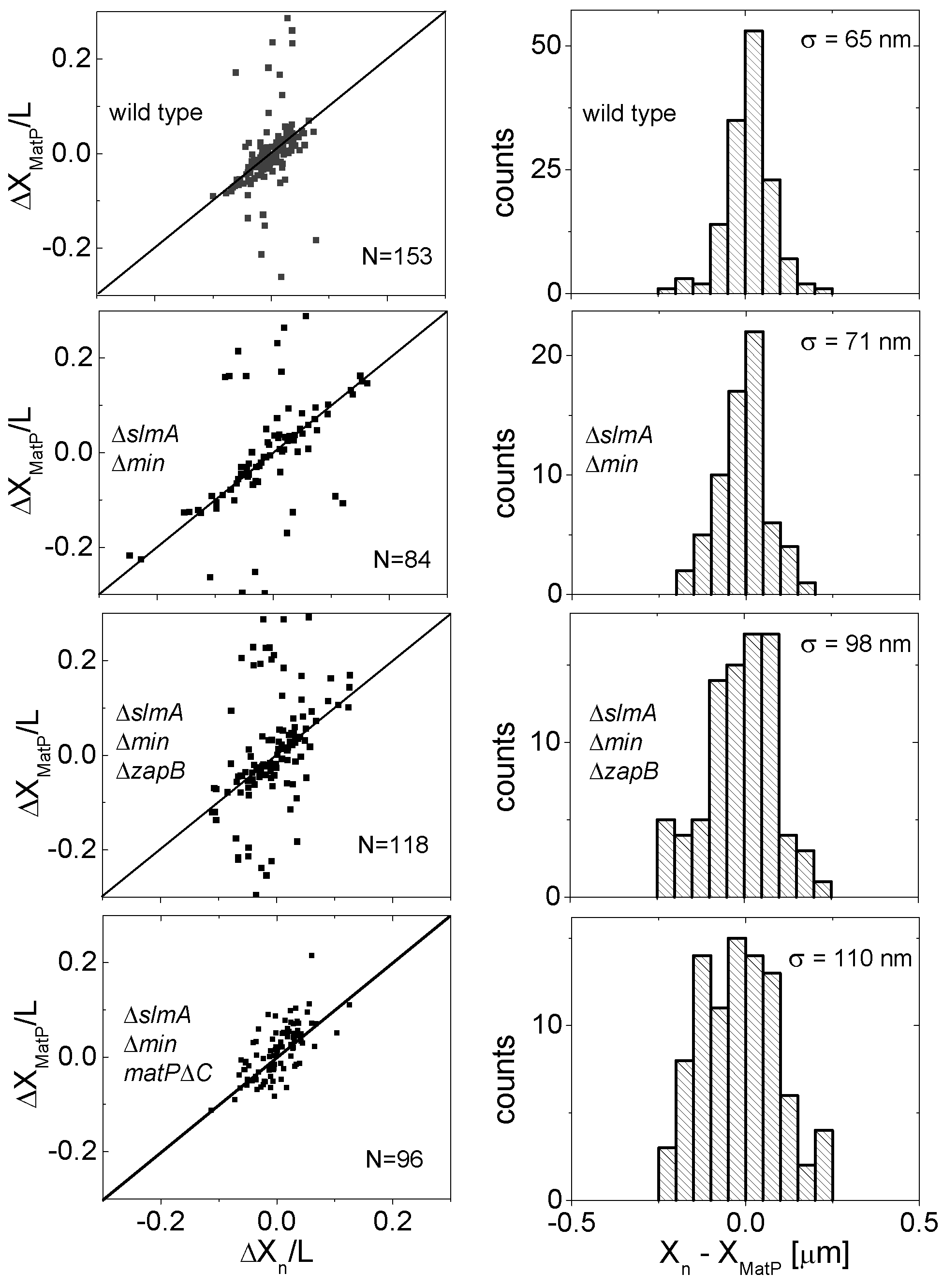

Supplement: Figure S14 — Left column: Displacements of MatP-focus relative to cell center, ΔXMatP, as a function of nucleoid displacement from cell center, ΔXn. All displacements are normalized by cell length L. The solid line corresponds to . Data are shown only for cells with a single nucleoid. The large scatter in ΔXMatP/L values in all strains is related to the movement of the Ter macrodomain from the nucleoid periphery to the center of the nucleoid early in the cell cycle. In the ΔslmA Δmin matPΔC strain, the movement of Ter macrodomain occurs before cell division. Consequently, in single nucleoid cells no MatP foci appear at the nucleoid periphery. Right column: Distance between nucleoid center and center of MatP focus. Each histogram is compiled from the data on the left column but retaining only these data where ΔXMatP is less than 0.25 µm from the nucleoid center. This selection eliminates spread caused by the cell cycle dependent movement of MatP focus from nucleoid periphery to nucleoid center. (TIF) [file pgen.1004504.s014.tif]
