# Supplementary material for: Evidence for Divisome Localization Mechanisms Independent of the Min System and SlmA in Escherichia coli
Source: PLoS Genet. 2014 Aug 7;10(8):e1004504. doi: 10.1371/journal.pgen.1004504 (PMC4125044; doi:10.1371/journal.pgen.1004504)
Supplement: Table S1 — List of the strains and plasmids used in experiments. (DOC) [file pgen.1004504.s016.doc]

**Table S1.** List of the strains and plasmids used in experiments.

| **Strain** | **Plasmid** | **Genotype** | **Source** |
| --- | --- | --- | --- |
| BW25113 | pKen1-GFPμ2 (1) | wild type | Keio collection parental strain (2) |
| JW5641-1 | - | *slmA*<>*frt*-*aph-frt* | Keio collection knockout (2) |
| JW1165 | - | *minC*<>*frt*-*aph-frt* | Keio collection knockout (2) |
| JW1165 | pKen1-GFPμ2 (1) | *minC*<>*frt*-*aph-frt* | Keio collection knockout (2) |
| PB194 | pKen1-GFPμ2 (1) | *slmA*<>*frt*-*aph-frt*  *minC*::*frt* | This work: kanamycin resistance cassette is evicted from Keio collection knockout JW1165 and resulting strain is P1 transduced with lysate from JW5641-1. |
| TB86(λCH151) | - | *slmA*<>*aph*  *ΔminCDE*<>*frt*  *P_lac_-zipA-gfp* | Gift from P. A. J. de Boer (Case Western Reserve University) (3) |
| TB86(λDR120) | - | *slmA*<>*aph*  *minCDE*<>*frt*  *P_lac_-gfp-T7tag-ftsZ* | Gift from P. A. J. de Boer (Case Western Reserve University) (3) |
| JMBW5 | - | *P_lac_-zipA-gfp* | This work: *zipA-gfp* was P1 transduced from TB86(λCH151) lysate to MG1655 strain. |
| WD1 | - | *slmA*<>*frt*  *minCDE*<>*frt*  *P_lac_-zipA-gfp*  *matP-mCherry-aph* | This work: kanamycin resistance cassette is evicted from TB86(λCH151).  C-terminal mCherry fusion to native *matP* |
| WD2 | - | *P_lac_-zipA-gfp*  *matP-mCherry-aph* | This work: JMBW5 was P1 transduced with *matP-mCherry* |
| MB4 | - | *slmA*<>*frt*  *minC*<>*frt*  *ΔzapB<> frt-aph-frt*  *P_lac_-zipA-gfp* | This work: kanamycin resistance cassette is evicted from PB194. The resulting strain is P1 transduced with *frt-aph-frt* lysate from Keio collection knockout JW3899-1 |
| MB10 | - | *slmA*<>*frt*  *minC*<>*frt*  *matP<>frt-aph-frt*  *P_lac_-zipA-gfp* | This work: kanamycin resistance cassette is evicted from PB194. The resulting strain is P1 transduced with *frt-aph-frt* lysate from Keio collection knockout JW0939-1 |
| MB11 | - | *slmA*<>*frt*  *minC*<>*frt*  *zapA<> frt-aph-frt*  *P_lac_-zipA-gfp* | This work: kanamycin resistance cassette is evicted from PB194. The resulting strain is P1 transduced with *frt-aph-frt* lysate from Keio collection knockout JW2878-1 |
| MB21 | - | *slmA*<>*aph*  *P_lac_-zipA-gfp* | This work: kanamycin resistance cassette is evicted from JW5641-1. The resulting strain is P1 transduced from TB86(λCH151) lysate to transfer *zipA-gfp*. |
| MB22 | - | *minC*<>*aph*  *P_lac_-zipA-gfp* | This work: kanamycin resistance cassette is evicted from JW1165. The resulting strain is P1 transduced from TB86(λCH151) lysate to transfer *zipA-gfp*. |
| MB25 | - | *slmA*<>*aph*  *P_lac_-zipA-gfp*  *matP-mCherry-aph* | This work: MB21 was P1 transduced with *matP-mCherry* |

| MB26 | - | *minC*<>*aph*  *P_lac_-zipA-gfp*  *matP-mCherry-aph* | This work: MB22 was P1 transduced with *matP-mCherry* |
| --- | --- | --- | --- |
| PB299 | - | *slmA*<>*frt-aph-frt*  *minC*<>*frt*  *zapB<> frt-cat-frt* | This work: PB194 is P1 transduced with *frt-cat-frt* lysate from HY1-32 (4). |
| PB300 | - | *slmA*<>*frt-aph-frt*  *minC*<>*frt*  *zapA<> frt-cat-frt* | This work: PB194 is P1 transduced with *frt-cat-frt* lysate from HY1-31 (4). |
| PB301 | - | *slmA*<>*frt-aph-frt*  *minC*<>*frt*  *matP<> frt-cat-frt* | This work: PB194 is P1 transduced with *frt-cat-frt* lysate from (5). |

<> denotes replacement

1. Cormack BP, Valdivia RH, Falkow S (1996) FACS-optimized mutants of the green fluorescent protein (GFP). *Gene* 173:33-38.

2. Baba T, Ara T, Hasegawa M, Takai Y, Okumura Y, Baba M, Datsenko KA, Tomita M, Wanner BL, Mori H (2006) Construction of *Escherichia coli* K-12 in-frame, single-gene knockout mutants: the Keio collection. *Mol. Syst. Biol.* 2:1-11.

3. Bernhardt TG, de Boer PAJ (2005) SlmA, a nucleoid-associated, FtsZ binding protein required for blocking septal ring assembly over chromosomes in *E. coli*. *Mol. Cell* 18:555-564.

4. Durand-Heredia JM, Yu HH, De Carlo S, Lesser CF, Janakiraman A (2011) Identification and Characterization of ZapC, a Stabilizer of the FtsZ Ring in *Escherichia* coli. *J. Bacteriol.* 193:1405-1413.

5. Mercier R, Petit M-A, Schbath S, Robin S, El Karoui M, Boccard F, Espeli O (2008) The MatP/matS Site-Specific System Organizes the Terminus Region of the *E. coli* Chromosome into a Macrodomain. *Cell* 135:475-485.
