# Supplementary material for: Evidence for Divisome Localization Mechanisms Independent of the Min System and SlmA in Escherichia coli
Source: PLoS Genet. 2014 Aug 7;10(8):e1004504. doi: 10.1371/journal.pgen.1004504 (PMC4125044; doi:10.1371/journal.pgen.1004504)
Supplement: Table S2 — Statistics describing co-localization of the Z-ring and the nucleoid center in different strains. R 2 is a dimensionless goodness of fit parameter for a model . Note that for perfect co-localization of nucleoid and the Z-ring centers, R2 approaches a value of one. R2 can also be negative; for there is no meaningful evidence of co-localization in the data. For cells with compact nucleoid morphology the percentage in parenthesis shows the frequency of those cells in the total population. Here, the total population accounts for all cells that have a single nucleoid and a single Z-ring. (DOC) [file pgen.1004504.s017.doc]

**Table S2.**

Statistics describing co-localization of the Z-ring and the nucleoid center in different strains.

*R*^2^ is a dimensionless goodness of fit parameter for a model Δ*X*_n_=Δ*X*_z_. Note that for perfect co-localization of nucleoid and the Z-ring centers, *R^2^* approaches a value of one. *R^2^* can also be negative; for *R*^2^<0 there is no meaningful evidence of co-localization in the data.

For cells with compact nucleoid morphology the percentage in parenthesis shows frequency of those cells in the total population. Here, the total population accounts for all cells that have a single nucleoid and a single Z-ring.

|  | Genotype | N | $\sigma_{X_{z}-X_{n}}$ (nm) | $\sigma_{X_{z}}$ (nm) | $R^{2}$ |
| --- | --- | --- | --- | --- | --- |
| JMBW5  all | Wild type | 202 | 81 | 118 | 0.51 |
| JMBW5  compact nucleoid | Wild type | 70  (25%) | 88 | 108 | 0.34 |
| MB21  all | Δ*slmA* | 166 | 63 | 78 | 0.54 |
| MB21  compact nucleoid | Δ*slmA* | 63  (34%) | 73 | 78 | 0.31 |
| MB22  all | Δ*minC* | 135 | 72 | 110 | 0.74 |
| MB22  compact nucleoid | Δ*minC* | 62  (20%) | 87 | 119 | 0.67 |
| TB86 ZipA  all | Δ*slmA* Δ*min* | 127 | 66 | 177 | 0.84 |
| TB86 ZipA compact nucleoid | Δ*slmA* Δ*min* | 74  (21%) | 95 | 178 | 0.84 |
| TB86 FtsZ  all | Δ*slmA* Δ*min* | 188 | 76 | 196 | 0.84 |
| TB86 FtsZ  compact nucleoid | Δ*slmA* Δ*min* | 55  (23%) | 81 | 195 | 0.82 |
| MB10  all | Δ*slmA* Δ*min* Δ*matP* | 123 | 151 | 200 | 0.40 |
| MB10  compact nucleoid | Δ*slmA* Δ*min* Δ*matP* | 50  (18%) | 230 | 230 | 0.04 |
| MB4  all | Δ*slmA* Δ*min* Δ*zapB* | 218 | 222 | 262 | 0.27 |
| MB4  compact nucleoid | Δ*slmA* Δ*min* Δ*zapB* | 51  (35%) | 344 | 352 | 0.11 |
| MB11  all | Δ*slmA* Δ*min* Δ*zapA* | 145 | 203 | 211 | 0.07 |
| MB11  compact nucleoid | Δ*slmA* Δ*min* Δ*zapA* | 50  (12%) | 280 | 275 | 0.01 |
