# Supplementary material for: Evidence for Divisome Localization Mechanisms Independent of the Min System and SlmA in Escherichia coli
Source: PLoS Genet. 2014 Aug 7;10(8):e1004504. doi: 10.1371/journal.pgen.1004504 (PMC4125044; doi:10.1371/journal.pgen.1004504)
Supplement: Table S3 — Frequency of polar Z-rings and minicelling divisions. (DOC) [file pgen.1004504.s018.doc]

**Table S3.**

Frequency of polar Z-rings and minicelling divisions.

| Genotype | % of minicelling divisions | N | % of  polar Z-rings | N |
| --- | --- | --- | --- | --- |
| Wild type | 0 | 943 | 0 | 202 |
| Δ*slmA* | 0 | 670 | 0 | 166 |
| Δ*minC* | 7 | 1490 | 16 | 160 |
| Δ*slmA* Δ*min* | 0.2 | 1326 | 14 | 155 |
| Δ*slmA* Δ*min* Δ*matP* | 28 | 660 | 69 | 394 |
| Δ*slmA* Δ*min* Δ*zapB* | 30 | 207 | 33 | 323 |
| Δ*slmA* Δ*min* Δ*zapA* | 8 | 422 | 52 | 245 |
