# Supplementary material for: Evidence for Divisome Localization Mechanisms Independent of the Min System and SlmA in Escherichia coli
Source: PLoS Genet. 2014 Aug 7;10(8):e1004504. doi: 10.1371/journal.pgen.1004504 (PMC4125044; doi:10.1371/journal.pgen.1004504)
Supplement: Text S1 — Description of image analysis algorithms to find positions of Z-rings, MatP, and the nucleoid centers relative to the cell center. (DOC) [file pgen.1004504.s022.doc]

**Text S1**

**Finding positions of Z-rings, MatP, and the nucleoid centers relative to the cell center.**

To analyze the tendency of the Z-ring to co-localize with the center of the bacterial nucleoid or with the MatP-labeled Ter macrodomain, the locations of the Z-ring, MatP focus, and nucleoid centers were determined relative to the geometric center of the rod-shaped cell. To find the cell center, a phase-contrast image of the cell was used. The center of the Z-ring, MatP focus, and the nucleoid were measured from fluorescent images of cells. The Z-ring was labeled with either ZipA-GFP or FtsZ-GFP, while the chromosomal terminus was observed using MatP-mCherry. The nucleoids were fluorescently imaged following DAPI-staining. Small sub-pixel level shifts between different images taken with GFP, RFP, and DAPI filtercubes, and phase contrast optics were corrected by shifting the phase contrast, RFP, and DAPI images relative to the GFP image or subtracting the shifts directly from measured coordinates.

The image analysis was carried out in Matlab making use of two publicly available libraries, DipImage Toolbox (http://www.diplib.org) and the PSICIC program (1). Analysis of phase-contrast images is based on the *Cload* function of PSICIC. *Cload* first defines the cell contour by grouping all pixels whose intensity values are less than a user-defined contour level, and subsequently determines the midline of each cell. The cell’s midline extends between cell poles following a path through the cell’s long axis. *Cload* finds the contour of the cell and its mid-line with sub-pixel resolution. We have made an addition to the PSICIC software to automatically determine the contour level thresholds for each cell. We define the contour level threshold as the averaged intensity value of all inflection points that surround the cell. The inflection points are calculated as zero crossings of the second derivative of the phase contrast image along the gradient direction (the direction of greatest intensity change). The calculation is based on the *laplace_plus_dgg* function from the DipImage library. The intensity values of all inflection points encircling each cell are averaged, and this averaged intensity value is passed to the *Cload* function. In addition to the cell contour, *Cload* returns a two-dimensional set of coordinates describing the midline of the cell. These coordinates are used to determine the geometrical center of the cell (as the midpoint of the midline), relative to which we determined the positions of the Z-ring, MatP focus, and the nucleoid. The midline is used also to calculate the length of the cell (L).

After defining the midline of each cell using *Cload*, the midline was broadened by constructing 4 additional, adjacent shifted curves to the midline (two on each side of the midline). The shift for each point was chosen perpendicular to the local tangent of the midline. The resulting broadened midline spans a total width of about 0.54 μm. Along each curve the fluorescence intensities were determined using cubic spline interpolation. The fluorescence intensities from different shifted curves were averaged in the perpendicular direction to the midline to define an effective intensity line profile along the long axis of the cell. The same procedure was carried out to yield ZipA-GFP, MatP-mCherry, and DAPI intensity distributions.

To determine the center of the Z-ring or the MatP focus, first the intensity distribution of ZipA-GFP or MatP-mCherry along the entire interpolated midline is plotted. From this extended profile, a region containing an intensity maximum, which corresponds to a Z-ring or a MatP focus, is manually selected. The selected region is then fitted to a Gaussian. The distance between the center of the Gaussian and the cell center (output by the *Cload* function) determines the distance ΔX_z_ or ΔX_MatP_, respectively.

To determine the geometrical center of the nucleoid, we plot the intensity profile of the DAPI-labeled nucleoid along the entire midline of the cell. We manually bracket the regions of the nucleoid profile containing the two outer-most inflection points of the nucleoid intensity distribution. These inflection points therefore define the edges of the nucleoid. Note that manual selection is necessary because more inflection points may exists in the intensity distribution from the DAPI-labeled nucleoid, especially when the nucleoids have started to segregate. The inflection points are determined using DipImage, which calculates the second derivative in the gradient direction of the fluorescent image. In each bracketed region, the second derivative of the intensity crosses zero only once. The particular midline coordinate of the inflection point is specified by interpolating linearly between the two coordinates in which the second derivative of intensity changes sign. The latter procedure yields the coordinates of the outermost edges of the nucleoid with sub-pixel resolution. The center of the nucleoid is defined as the midpoint between the coordinates of the two outer edges (the inflection points) of the nucleoid. The distance between the center of the nucleoid and the cell center, as found previously with *Cload* function, determines distance ΔX_n_.

1. Guberman JM, Fay A, Dworkin J, Wingreen NS, Gitai Z, (2008) "PSICIC: Noise and Asymmetry in Bacterial Division Revealed by Computational Image Analysis at Sub-Pixel Resolution", PLoS Comput. Biol. 4 e1000233.
